# Sublethal Effects and Associated Risks of Acaricides Used Against *Varroa destructor* in Honey Bee (*Apis mellifera*) Colonies

**DOI:** 10.3390/insects17050517

**Published:** 2026-05-19

**Authors:** Louise Petit, Cameron J. Jack

**Affiliations:** Honey Bee Research and Extension Laboratory, Entomology and Nematology Department, University of Florida, Gainesville, FL 32611, USA; cjack@ufl.edu

**Keywords:** acaricides, *Apis mellifera*, honey bee, toxicology, *Varroa destructor*

## Abstract

Beekeepers widely recognize *Varroa destructor* (Mesostigmata: Varroidae) as one of the most serious threats to honey bee (*Apis mellifera*) health. To control *V. destructor*, beekeepers routinely use chemical treatments that reduce mite populations, benefiting colonies. However, these treatments also expose honey bees to compounds that may cause unintended harm. Honey bees and *V. destructor* share biological structures that are targeted by acaricides. In this review, we describe the main modes of action of commonly used synthetic and natural chemicals and discuss differences between mites and honey bees that explain selectivity. In addition, exposure to non-lethal doses can potentially disrupt honey bee functions outside the treatment’s primary targets. Here, we synthesize current knowledge on the effects of *V. destructor* control chemicals on honey bee health at the molecular level, the individual level (including larvae, workers, queens, and drones), and the colony level. Unfortunately, not all tissues and colony functions have been studied for every compound. Therefore, this review also identifies knowledge gaps where the effects are still unknown. We believe that a better understanding of the effects of *V. destructor* treatments on honey bee colonies will help beekeepers manage these treatments more carefully, benefiting both beekeepers and honey bees.

## Simple Summary

Pesticides and veterinary drugs are approved for use with the assumption that their benefits outweigh their risks. In the beekeeping world, beekeepers use treatments to control pests and pathogens, mainly *Varroa destructor* (Mesostigmata: Varroidae), an ectoparasitic mite that is one of the major threats to honey bee (*Apis mellifera*) colonies. Common synthetic treatments to control *V. destructor* belong to three main acaricide groups: pyrethroids (*tau*-fluvalinate and flumethrin), organophosphorothioates (coumaphos), and formamidines (amitraz). Natural treatments are also used and include organic acids such as formic acid and oxalic acid, as well as essential oil compounds, such as thymol. Beekeepers routinely apply these chemical treatments to reduce *V. destructor* populations, which benefits colonies by lowering mite pressure. These products are authorized based on the assumption that the risks to honey bees are lower than the benefits of mite control. However, some negative effects, especially long-term ones, may only become apparent after extensive use. In this review, we use published studies to determine the sublethal effects of commonly used *V. destructor* control compounds on honey bee health. We focus on effects at different levels of biological organization, from molecular and individual effects to impacts on colony health.

## 1. Introduction

*Varroa destructor* (Mesostigmata: Varroidae) is widely recognized by beekeepers as one of the most significant threats to honey bee health [1]. Research supports this view, with decades of research demonstrating the impact that *V. destructor* has on honey bee colony losses [2,3,4,5]. Infestation by these mites weakens honey bee colonies, often leading to their demise within three or four seasons if no treatment is applied [6,7,8].

*V. destructor* is a natural parasite of the eastern honey bee (*Apis cerana*). However, introduction by humans of the western honey bee (*Apis mellifera*) into regions in Asia occupied by the *Apis cerana* allowed the mite to shift hosts in the 1950’s [3]. From there, *V. destructor* spread via natural bee movements like swarming and through human activities, such as commercial pollination and the shipment of bees. The mite was first detected in Europe in the 1960s (Bulgaria, 1967) and in the Americas in the 1970s (Paraguay, 1971) [3]. Now, *V. destructor* has a worldwide distribution and can be found on all continents where *A. mellifera* is kept [9]. Colonies with high mite loads grow their worker population more slowly and are weakened [10]. At the hive entrance, one can find many dead or lethargic adult honey bees [11], and a high mite infestation level may trigger the hygienic removal of infested pupae [12]. Furthermore, many impacts of varroosis are directly related to the viruses transmitted by the mite during infestation [13].

**Figure 1:**
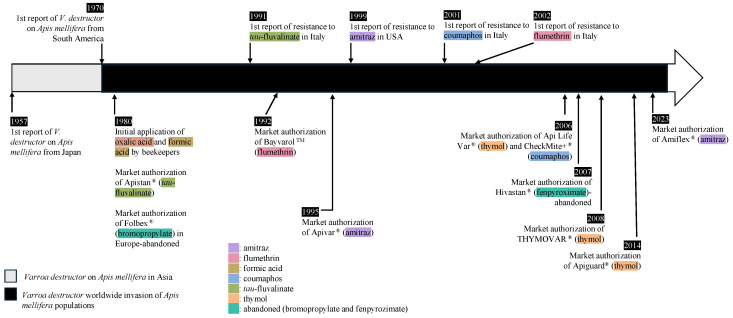
Timeline of *Varroa destructor* invasions and market authorization for acaricides to treat *V. destructor* in honey bee colonies. Sources: U.S. Environmental Protection Agency/European Medicines Agency regulatory records [3,15,16,18].

By weakening honey bee colonies, *V. destructor* also undermines the activity of beekeepers worldwide. In response to the threat of *V. destructor*, various treatments were developed to manage mite infestations. Since the 1980s, beekeepers have used ‘soft chemicals’ like formic acid and oxalic acid to control *V. destructor* [14]. However, the use and efficacy of natural compounds are highly variable and typically short-lasting, so synthetic chemical treatments provide longer-lasting effects. In 1980, Apistan^®^ (*tau*-fluvalinate, pyrethroid) was introduced in Europe, but by the mid-1990s, reports of *V. destructor* developing resistance to this compound were noted [15]. Around the same time, another treatment from a different pesticide class, CheckMite+^®^ (coumaphos, organophosphorothionate), was also introduced, but resistance to it emerged in 2001 [16]. One major concern associated with these two synthetic chemicals is their lipophilic nature, which causes them to accumulate in beeswax over time [17]. This chemical accumulation poses risks not only to honey bees but also potentially to beekeepers and consumers. In 1995, Apivar^®^ (amitraz, formamidine) was introduced in Europe, but resistance was reported as early as 1999 [18]. Over the past few decades, there has been an increasing demand for treatments compatible with organic honey production. In response, thymol-based treatments such as Api Life Var^®^ (thymol) were introduced in 2006, and several other thymol-based products have been developed since (Figure 1).

This review focuses on active ingredients and commercial formulations that are registered and used in North America and Europe. Specifically, it includes amitraz, coumaphos, *tau*-fluvalinate, flumethrin, formic acid, oxalic acid, and thymol. While other acaricides and treatments are used in different parts of the world, they are not reviewed here.

While these acaricides help control mite infestations, they are not without risks. Each treatment carries potential hazards for honey bees, beekeepers, and consumers. These veterinary treatments were approved based on the assumption that their benefits outweighed the risks. However, some adverse effects, especially long-term ones, may only become apparent after extensive use. This review will explore the risks associated with the use of acaricides for *V. destructor* control and their potential impacts on honey bee colonies.

## 2. Methodology

We performed a literature search to identify studies on the sublethal effects of acaricides used to control *V. destructor* in *Apis mellifera* colonies. This review covers acaricides that are registered and used in North America and Europe. Namely, amitraz, coumaphos, *tau*-fluvalinate, flumethrin, formic acid, oxalic acid, and thymol.

Studies were found using multiple databases, including Google Scholar and PubMed. The reference lists of relevant articles were also screened to identify additional studies. In addition, Connected Papers (based on Semantic Scholar) was used to identify additional relevant publications.

The search covered studies published approximately from 1980 (the period marking the development and introduction of acaricides for *V. destructor* control) to December 2025. Searches were performed using the following keywords: “*Varroa destructor*”, “*Apis mellifera*”, “honey bee”, “acaricides”, “miticides”, “amitraz”, “coumaphos”, “*tau*-fluvalinate”, “flumethrin”, “formic acid”, “oxalic acid”, and “thymol”.

Studies were included if they investigated the effects of acaricides used for *V. destructor* control, specifically focusing on the compounds listed above. All formulations and commercial product names were considered. Peer-reviewed articles published in English were included. Moreover, studies identified using English keywords but written in other languages were also considered when relevant data were available.

In addition, theses, dissertations, and conference posters were included when they provided relevant data. These sources were incorporated to capture emerging or unpublished findings relevant to this review. All studies, regardless of publication type, were assessed using the same inclusion criteria. When multiple studies reported different or conflicting results, all findings were presented and discussed.

## 3. Mode of Action of Chemicals Used to Control *Varroa destructor* and Their Products

### 3.1. Organic Chemicals

Many beekeepers use natural compounds to control *V. destructor,* often referred to as ‘soft’ treatments [14]. This choice is commonly motivated by the belief that these compounds are less harmful to honey bees and the environment than synthetic chemicals. Organic chemicals include organic acids such as formic acid and oxalic acid, as well as essential oil compounds, such as thymol. Each of these chemicals has a different mode of action in killing *V. destructor.* Understanding their modes of action is essential for predicting their toxicity to honey bees. Herein, we will discuss treatments registered in North America and Europe; however, other products, formulations, and active ingredients are used in other parts of the world.

#### 3.1.1. Formic Acid

Formic acid is a natural compound commonly found in ant venom [19]. While its mode of action is not yet fully understood, some research indicates that formic acid inhibits electron transport in the mitochondria of insects by binding to cytochrome c oxidase [20]. Additionally, this binding can lead to significant neuroexcitatory effects [21]. Formic acid is the only treatment for *V. destructor* that effectively targets both phoretic mites and reproductive mites contained within capped brood cells [22]. It can be found in products such as Formic Pro^TM^ (NOD Apiary Products Ltd., Quinte West, ON, Canada), Mite-Away Quick Strips^®^ (NOD Apiary Products Ltd., Quinte West, ON, Canada), and VarroMed^®^ (BeeVital, Vienna, Austria).

#### 3.1.2. Oxalic Acid

Oxalic acid is a natural compound that is present in the metabolic reactions of both plants and animals [23]. While its mode of action against *V. destructor* is not yet fully understood, researchers have found that oxalic acid kills *V. destructor* upon contact [24]. Like most chemical treatments, oxalic acid is most effective during broodless periods while mites are on the bodies of adult bees, as it cannot kill mites that are inside capped cells [25]. It can be found in products such as Oxybee^®^ (Veto-Pharma, Palaiseau, France), Api-Bioxal RTU beehive solution^®^ (Chemicals Laif S.P.A., Vigonza, Italy), EZ-OX Tablets™ (Mike’s Bees LLC, Cambridge, MA, USA), VarroxSan™ (Vita Bee Health Limited, Basingstoke, UK), and VarroMed^®^ (BeeVital, Vienna, Austria).

#### 3.1.3. Essential Oil Compound

Thymol is an essential oil compound authorized for the treatment of *V. destructor* in honey bee colonies in North America and Europe. Thymol is a major component of *Satureja satureioides* and *Lippia gracillis*. Thymol affects the insect neurosystem by modulating GABA-activated ion channels, especially insect RDL receptors. Researchers have shown that RDL receptors in honey bees and *V. destructor* show different responses to thymol, which explains why this essential oil compound is less toxic to honey bees than to the mite [26]. Moreover, researchers have demonstrated that thymol can inhibit acetylcholinesterase (AChE) activity [27], affect Transient Receptor Potential (TRP) channels [28], block the binding of [^3^H]-tyramine to tyramine receptors [29], and reduce flight muscle impulse and wing beat frequency [30] in insects. It can be found in products such as Apiguard^®^ (Vita Europe, Basingstoke, UK), Api Life Var^®^ (Chemicals LAIF S.P.A., Vigonza, Italy), and THYMOVAR^®^ (Andermatt, Grossdietwill, Switzerland).

### 3.2. Synthetic Chemicals

Four synthetic chemicals are currently approved for the treatment of *V. destructor* in honey bee colonies in North America and Europe: *tau*-fluvalinate, flumethrin, coumaphos, and amitraz. These chemicals belong to three classes of acaricide: pyrethroids (*tau*-fluvalinate and flumethrin), organophosphothionates (coumaphos), and formamidines (amitraz).

#### 3.2.1. Formamidines

**Figure 2:**
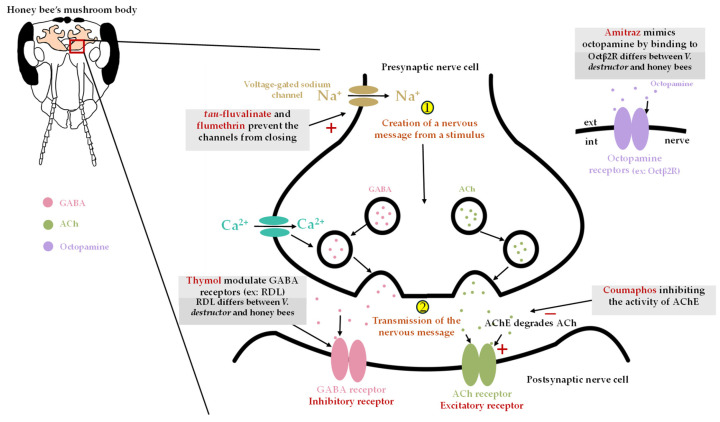
Known mode of action of principal chemicals used to control *V. destructor* and their potential negative effects on the honey bee nervous system. Abbreviations: AchE, acetylcholinesterase; GABA, gamma-aminobutyric acid; RDL, resistant to dieldrin; Ach, acetylcholine; Octβ2R, octopamine beta 2 receptor.

Amitraz is a common formamidine acaricide that targets the nervous system of pests. This acaricide is unstable and rapidly degrades into two major metabolites: 2,4-dimethylformamide (DMF) and N-(2,4-dimethylphenyl)-N’-methylformamidine (DMPF). Amitraz mimics the actions of octopamine, a neurohormone that regulates a range of physiological processes in insects by binding to octopamine receptors [31] (Figure 2). In honey bees, octopamine plays a role in olfactory learning and memory, feeding response, vision, division of labor, dance behavior, discrimination of nestmates from non-nestmates and sting responses [32]. In *V. destructor*, the receptors Oamb, Oct-TyrR, OctαR, and Octβ2R are all activated by amitraz and DMPF [33]. Among these, the Octβ2R receptor appears to be the primary mediator of amitraz toxicity to pests. This receptor differs between *V. destructor* and honey bees, explaining why amitraz is less toxic to honey bees than to the mite [33]. Overstimulation of octopamine receptors leads to paralysis, tremors, and death in the affected insects. Amitraz is the active ingredient found in treatments such as Apivar^®^ and Amiflex^®^ (Veto-pharma, Palaiseau, France).

#### 3.2.2. Organophosphothionates

Coumaphos is an organophosphothionate and acaricide that targets the nervous system of insects and mites. It acts by inhibiting the activity of acetylcholinesterase (AchE), which prevents the breakdown of acetylcholine at synapses [34] (Figure 2). This leads to an accumulation of acetylcholine, resulting in overstimulation of the nervous system. Affected insects experience tremors, convulsions, hyperexcitability, and eventually paralysis, leading to death. The active ingredient of Checkmite+^®^, once manufactured by Bayer, now by Elanco, includes coumaphos as an active ingredient.

#### 3.2.3. Pyrethroids

Flumethrin and *tau*-fluvalinate are pyrethroid acaricides that also target the nervous system of mites. They act by binding to voltage-gated sodium channels in nerve cells [35] (Figure 2). These compounds prevent the channels from closing, leading to a continuous influx of sodium into the nerve. This overstimulation of the nerve causes tremors, hyperactivity, convulsions and paralysis that lead to death [36]. Flumethrin is the active ingredient of Bayvarol^®^ (Elenco, Indianapolis, IN, USA) and *tau*-fluvalinate is the active ingredient of Apistan^®^ (Vita Bee Health, Basingstoke, UK).

## 4. Existing Treatments Used to Control *Varroa destructor*: Dosage, Duration, and Timing of Application

Treatments available for *V. destructor* control vary in terms of active ingredient quantity, treatment duration, application method, and timing (Table 1). Each of these parameters can influence the toxicity of chemicals for the colony.

- Quantity of active ingredients: The toxicity of a chemical is linked to the quantity to which honey bees are exposed. Many acaricides are neurotoxic to insects, meaning that a low dose has an impact on a small number of targets and can lead to subtle changes in memory, learning and behavior, for example [37,38]. Whereas higher doses will have an impact on a larger number of targets, leading to death.
- Duration of treatment: Longer treatment times expose honey bees to chemicals for longer periods. Continuous or repeated exposure can result in cumulative toxicity, weakening bees over time, even if each individual exposure is below lethal levels [39]. Short-term exposure, on the other hand, can have more immediate effects.
- Method of application: The way the chemical is introduced to the bees influences its impact [40]. For example, contact treatments may be in contact only with the areas of the body in contact with the substance. In contrast, methods like sublimation or fumigation can enter the honey bee’s respiratory system, leading to a different response [41].
- Timing of treatment: The colony activity changes with the seasons, and the timing of chemical application can influence its impact [42]. For example, in spring, when drones are produced for mating and queens are preparing for reproduction, exposure to acaricides can have an impact on reproductive processes [43,44]. On the other hand, in early autumn, when the drones are expelled from the hive and die, the impact of acaricide exposure on the colony for this caste may be low.

**Table 1:** Dosage, duration, and timing of application for products used by beekeepers to control *V. destructor* in hives (according to labels).

Commercially Available Products	Chemical	Application	Quantity of Active Ingredient per Treatment (g)	Treatment Duration (Weeks)	Treatment Prerequisites
Oxybee^®^	Oxalic acid, anise, and eucalyptus oils	Spray, trickling and sublimation	1.97	<1	Colony without brood Absence of honey
Api-Bioxal RTU beehive solution	Oxalic acid	Spray, trickling and sublimation	1–3	<1	Colony without brood
VarroxSan^TM^	Oxalic acid	Strips	28	6	One chamber separates the treatment of any honey to be extracted
EZ-OX Tablets	Oxalic acid	Tablets	2–4	<1	Colony without brood
Formic Pro^TM^	Formic acid	Strips	136	2	Colony with brood, temperature between 10–29 °C
VarroMed^®^	Oxalic acid/ formic acid	Spray, trickling and sublimation	0.05 formic acid/0.04 oxalic acid	4	Absence of honey
Apiguard	Thymol	Gels	12.5	6	Temperature between 15–40 °C
Api Life Var	Thymol/oil of eucalyptus/menthol	Plates	8 thymol/1.72 oil of eucalyptus/0.32 menthol	4	Temperature under 35 °C
THYMOVAR^®^	Thymol	Strips	15	4	Temperatures under 30 °C
APISTAN^®^	*tau*-fluvalinate	Strips	1.65	6	Absence of honey
Apivar^®^	Amitraz	Strips	1	6	Absence of honey
Amiflex^®^	Amitraz	Gels	0.13	1	Absence of honey
CheckMite+^®^	Coumaphos	Strips	1.36	6	Absence of honey
Perizin^®^	Coumaphos	Trickling	0.03	6	Absence of honey
Bayvarol^TM^	Flumethrin	Strips	0.01	6	Absence of honey

## 5. Impact of Acaricides at the Molecular Level

**Figure 3:**
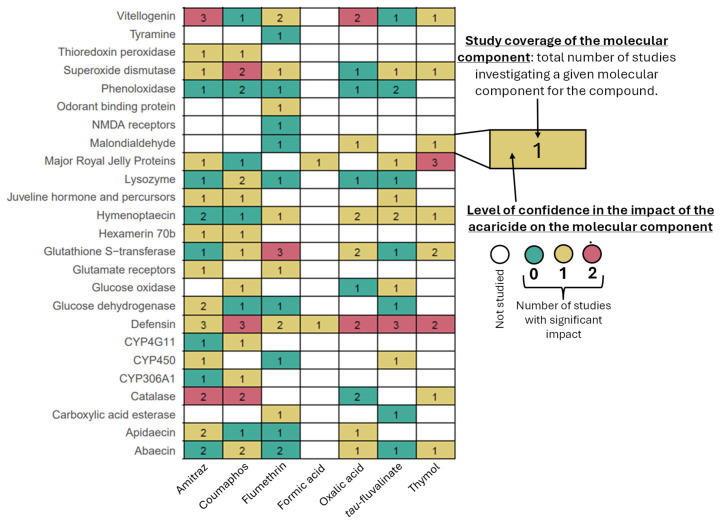
Impact of acaricides to control *V. destructor* on honey bees at the molecular level. The number shown in each box corresponds to the total number of published research studies investigating the molecular component of the compound. The color of the box indicates the level of confidence in the observed impact. Green indicates that no study has found a significant impact. For negative impacts, the color is yellow when only one study reports a significant effect, and red when two studies report a significant effect.

This section presents studies investigating the impact of each compound on honey bee molecular components. Some molecular components have not been studied for all of the compounds. These gaps are shown in Figure 3 and Table S1 and are further discussed in the conclusion.

Researchers have investigated the impact of acaricides on honey bees at the molecular level, but the results are quite varied (Table S1). Across studies, significant increases or decreases compared to the control have been reported in genes and proteins involved in immunity, detoxification, development, stress, the nervous system, and the olfactory system (Table S1) [45,46,47,48,49,50,51,52,53,54,55,56,57,58,59,60,61,62,63,64,65,66]. However, responses are rarely consistent, even for the same compound.

For instance, studies on vitellogenin expression after amitraz exposure have shown varying results. While one study described no impact [45], another observed a decrease [46], and a third reported an increase [47]. These discrepancies likely arise due to differences in honey bee genetics, exposure methods, and other environmental factors.

Honey bee cells can be cultured in vitro. Researchers in one study showed that exposing honey bee cells to thymol in the laboratory caused DNA damage, raising questions about what the effects might be on honey bees [48]. Another study showed a reduction in DNA damage after oral exposure of colonies to thymol (0.1 g/L) [49].

## 6. Impact of Acaricides at the Tissue Level

**Figure 4:**
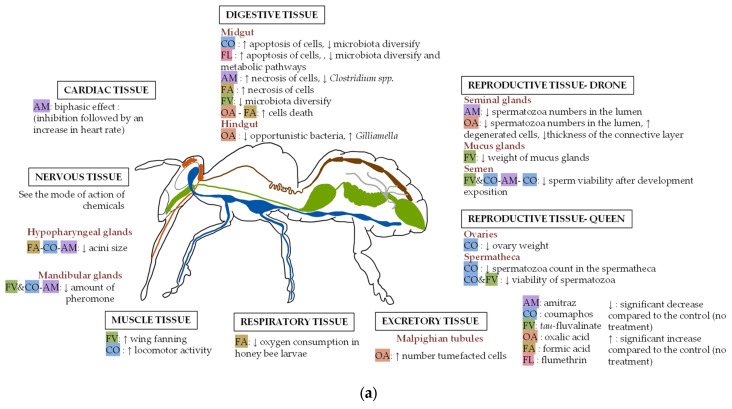

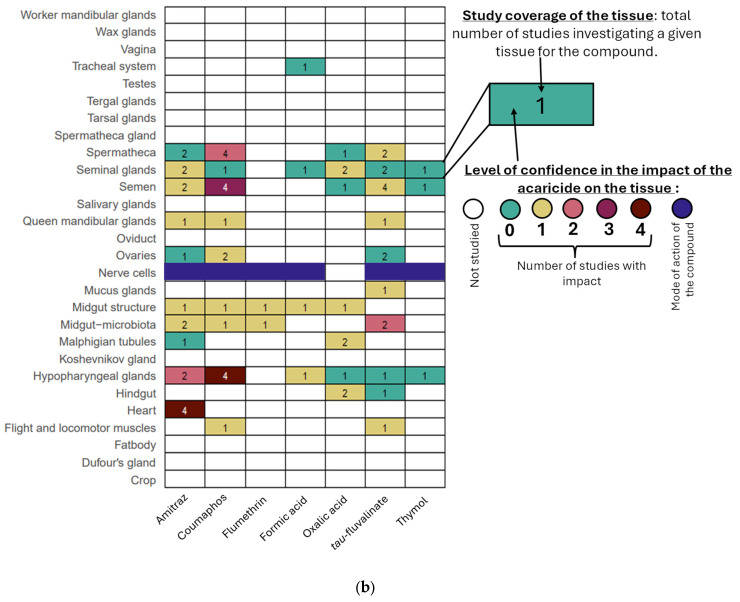
Impact of acaricides to control *V. destructor* on honey bees at the tissue level. (**a**) Diagram illustrating the significant sublethal effects of acaricides on organs of adult honey bees, as described in the peer-reviewed scientific literature. (**b**) Figure describing the impact of acaricides on each tissue of adult honey bees. The number shown in each box corresponds to the total number of studies investigating the tissue for the compound. The color of the box indicates the level of confidence in the observed impact. For amitraz, studies showing the effects of its metabolites were also included in this figure. Moreover, for studies that reported impacts from combinations of two acaricides, a negative effect was assigned to each compound as a precautionary approach.

This section presents studies investigating the impact of each compound on honey bee tissues. Some tissues have not been studied for any of the compounds. These gaps are shown in Figure 4 and are further discussed in the conclusion.

### 6.1. Muscle Tissue

Amitraz has been shown to have cardiotoxic effects on honey bees (Figure 4). Researchers have indicated that it may first cause a reduction in heart rate, followed by an acceleration [67,68]. This biphasic effect is thought to result from amitraz acting as an octopamine mimic, as octopamine in honey bee physiology similarly causes an initial phase of inhibition followed by an increase in heart rate [69]. In addition, one of amitraz’s metabolites, DMPF, which can remain in the hive for a much longer period, appears to produce the same cardiotoxic effects [67].

*tau*-fluvalinate and coumaphos have been shown to have a negative impact on honey bee muscle activity, affecting locomotion. *tau*-fluvalinate caused honey bees to perform significantly longer wing fanning behaviors compared to honey bees not exposed to this compound [70]. This effect may be attributed to a disruption in the wing muscle function, as pyrethroids have been shown to disrupt tight coupling between right and left muscles in the house fly *Musca domestica* [71]. Coumaphos has been found to increase honey bee locomotor activity [72]. Although researchers have yet to examine the direct impact of amitraz on honey bee locomotion, research on octopamine showed that this compound influences insect mobility [73], suggesting that amitraz could have similar effects. All of these muscular effects may result from disruption of the nervous system, as muscle activity is regulated by neural inputs.

### 6.2. Nervous Tissue

The impact of acaricides on honey bee nervous tissue relates to the mode of action of these acaricides (Figure 2), as they act by targeting the nervous system. Honey bees exposed orally to 12.5–50 ppm thymol did not exhibit an increase in the amount of apoptosis in brain tissue [63].

### 6.3. Digestive Tissue

#### 6.3.1. Midgut and Hindgut

1. Midgut: The honey bee midgut is the central part of its digestive system, functioning similarly to the stomach in other animals. Situated between the crop and the hindgut, it plays a role in nutrient absorption and digestion. Researchers have shown that amitraz, coumaphos, flumethrin, oxalic acid, and formic acid all can alter the midgut’s histological structure (Figure 4). For example, coumaphos-treated honey bees experience significant apoptosis in midgut tissue [74], as do those treated with flumethrin [75]. Necrosis has also been observed in honey bee midguts following amitraz [76] and formic acid [77] treatments. Furthermore, oxalic acid appears to increase cell death in the midgut [77,78]. Acaricides used for *V. destructor* control also impact the honey bee’s midgut microbiota. Amitraz, for instance, has shown minimal effects on honey bee microbiota, with studies indicating no significant changes in intestinal communities [79]. Another study, however, noted a minor change, including reduced levels of *Escherichia coli* and particularly *Clostridium* spp., and a decline in fungal communities [80]. The microbiota response to amitraz appears to be method dependent and suggests greater sensitivity under in-hive exposure conditions than under controlled laboratory exposure. However, it remains difficult to determine whether these changes to the microbiota are biologically meaningful. In contrast, flumethrin disrupts intestinal function by decreasing microbiota diversity and altering metabolic pathways [52]. *tau*-fluvalinate similarly affects microbiota composition, increasing the presence of *Snodgrassella* and *Zygosaccharomyces* while reducing *Commensalibacter* in worker bees [81]. In field conditions, both coumaphos and *tau-*fluvalinate exposure significantly affected bacterial community structure but did not alter fungal communities [82].
2. Hindgut: The honey bee hindgut is a part of the digestive tract that includes the ileum and rectum. One study assessed the impact of *tau*-fluvalinate on the hindgut microbiota (Figure 4), finding no statistically significant differences in the relative abundance of *Lactobacillus* spp., *Commensalibacter* spp., *Serratia* spp., and *Snodgrassella* spp. [79]. Another study demonstrated that oxalic acid treatment in colonies caused a disrupted gut microbiota, reducing bacterial diversity by decreasing the presence of opportunistic bacteria. This treatment also enriched beneficial bacteria, such as *Gilliamella*, in both the hindgut and midgut of the honey bees [83]. Oxalic acid exposure also caused severe degeneration of the rectal epithelium within 48 h after treatment [84]. These effects may depend on the application method, particularly when oxalic acid is administered via trickling in a sucrose solution, as in the case of Martín-Hernández et al. [84], which can stimulate grooming behavior and increase ingestion, potentially increasing exposure of the hindgut. The observed enrichment of beneficial bacteria may also correspond to a microbiota recovery after initial disruption caused by the treatment.

#### 6.3.2. Malpighian Tubules

Malpighian tubules are the excretory organs of insects and play a crucial role in osmoregulation. At a concentration of 1320 µg oxalic acid per bee, oxalic acid affects the physiology of Malpighian tubules, causing the swelling of cells [84] (Figure 4). However, at lower doses, oxalic acid appears to have no impact on the physiology of these organs [85]. Regarding synthetic chemicals, the impact of the amitraz metabolite DMPF on honey bee Malpighian tubules was studied. The researchers observed that, at field-relevant concentrations, there is no significant difference in the number of normal and darkened Malpighian tubules compared with controls [86].

### 6.4. Glandular Tissue

#### Hypopharyngeal and Mandibular Glands

The hypopharyngeal glands are paired structures located bilaterally in the head of worker honey bees, in front of the brain between compound eyes. These glands are crucial for producing larval diets, with secretions that are clear and rich in proteins. Researchers have shown that coumaphos, amitraz and formic acid negatively affect the size of hypopharyngeal glands in honey bees (Figure 4). Coumaphos seems to hypertrophy acinis in hypopharyngeal glands [87,88,89], possibly as a result of gland necrosis [90]. Additionally, amitraz reduces the acini diameter and perimeter [91] and leads to the presence of fine basophilic granules in the cytoplasm [92]. *tau*-fluvalinate appears to have no significant effect on cell size or nucleus size in the glands [87]. However, formic acid does appear to atrophy acini cells and often causes the absence of nuclei [92]. Thymol (0.5%, 3%, and 6%) and oxalic acid (0.5%, 3%, and 6%) were shown to have no impact on the development of hypopharyngeal glands after feeding [93].

In worker bees, the mandibular glands also contribute to larval diet production, releasing a white, lipid-rich secretion. However, to our knowledge, no studies have yet examined the effects of acaricides on these other larval diet glands.

### 6.5. Respiratory Tissue

Honey bees possess thin-walled air sacs distributed at various places for respiration. Air exchange with the external environment occurs through 10 pairs of openings called spiracles. Formic acid, oxalic acid, and thymol are treatments that act by dispersing into the hive atmosphere. One study showed that airborne formic acid inhibited oxygen consumption in honey bee larvae after 120 h’ exposure [41] (Figure 4). However, no necrosis or corrosion of the honey bee’s tracheal system was observed [41]. No studies have examined the impact of synthetic chemicals on the respiratory system of honey bees.

### 6.6. Endocrine Tissue

#### Mandibular Glands

In queens, mandibular glands produce pheromones that help maintain colony organization as well as social learning and cohesion [94]. Exposure to acaricides such as amitraz and a mixture of coumaphos and *tau*-fluvalinate, during development, has been shown to reduce the attractiveness of queen mandibular gland contents to workers by ~12% for amitraz and 20% for a *tau*-fluvalinate and coumaphos mixture and affect the amount of the gland’s pheromone [95] (Figure 4). This result suggests that these compounds can impair queen recognition and social cohesion within the colony.

### 6.7. Reproductive Tissues

#### 6.7.1. Queen Ovaries and Spermatheca

Researchers have observed that acaricides generally do not affect ovary weight or sperm viability in the spermatheca of honey bee queens, although some studies suggest that coumaphos may impact both (Figure 4).

1. Ovaries: Queen honey bees have two large, active ovaries capable of producing around 2000 eggs per day [96]. Queens reared in beeswax impregnated with amitraz or a combination of *tau*-fluvalinate and coumaphos show no change in the number of ovarioles per ovary [97]. However, exposure via beeswax may not accurately reflect actual chemical exposure, as not all compounds are equally bioavailable to developing bees. Similarly, colonies treated with *tau*-fluvalinate exhibit no significant differences in queen ovary weight compared to controls. However, colonies exposed to coumaphos demonstrate a significant reduction in ovary weight [98].
2. Spermatheca: The spermatheca is a structure in queens that stores sperm and allows the queen to control which eggs are fertilized. Regarding queen spermatheca, no significant differences in sperm viability were observed in queens reared in amitraz-impregnated wax [99] or exposed to 2.0 ppm of amitraz in laboratory conditions [46]. Likewise, queens exposed to *tau*-fluvalinate or coumaphos during development via beeswax show no significant impact on sperm viability [99]. Queens from colonies treated with oxalic acid also exhibit no differences in sperm viability [100]. Honey bee queens topically treated with coumaphos at concentrations from 0.02 ppm to 0.4 ppm show no significant impact on sperm viability [50]. In contrast, queens reared in beeswax containing 100 mg/kg of coumaphos display a decrease in the percentage of queens with a clear or white spermatheca, indicating a reduction in spermatozoa count [101]. Additionally, queens reared in cups containing a combination of *tau*-fluvalinate and coumaphos showed a reduction in sperm viability in the spermatheca [102].

#### 6.7.2. Drone Reproductive Organs

1. Seminal glands: In honey bees, spermatozoa are transferred from the testes to the seminal vesicles as the drone matures. The seminal vesicles play a role in maintaining sperm quality (viability and motility) until the drone mates with the queen. Proteins produced by the seminal gland cells help to preserve the quality of spermatozoa both before and after mating [103]. Several studies have investigated the impact of acaricides on seminal gland size (Figure 4). One study reported that *tau*-fluvalinate did not significantly affect seminal vesicle weight [104] and another found that coumaphos had no impact on seminal vesicle length and width [105]. Researchers have also focused on acaricide effects on spermatozoa count in the seminal vesicle lumen. Researchers in one study observed a significant reduction in spermatozoa numbers following exposure to amitraz but found no differences for *tau*-fluvalinate, oxalic acid, formic acid, or thymol [106]. Another group reported a significant decrease in spermatozoa with oxalic acid exposure, but found no effects for amitraz [107]. Here, researchers also detected degenerated cells in drones exposed to oxalic acid and a notable reduction in the thickness of the connective layer, though not in the muscle layer [107]. These observations suggest that oxalic acid exposure may impair sperm quality.
2. Mucus glands: Mucus glands produce seminal fluid, which affects sperm viability in the queen’s reproductive tract [103]. One study found that *tau*-fluvalinate significantly reduced the weight of the mucus glands [104] (Figure 4).
3. Semen: Honey bee drone semen is composed of spermatozoa and a seminal fluid. During mating, the drone inserts his endophallus into the queen’s vagina, depositing the semen. Ref. [44] found no significant impact on sperm viability in drone semen when exposed to amitraz, coumaphos, oxalic acid, thymol, or *tau*-fluvalinate via the hive (Figure 4). However, another study observed no significant effect of *tau*-fluvalinate on sperm viability but reported a significant reduction after exposure to coumaphos [108]. Additionally, when drones were developed in beeswax contaminated with amitraz or a combination of *tau*-fluvalinate and coumaphos, sperm viability significantly decreased [43]. Another study further confirmed a significant reduction in sperm viability after six weeks of coumaphos exposure during drone development, a result not observed with *tau*-fluvalinate [108].

## 7. Impact of Acaricides at the Individual Level

**Figure 5:**
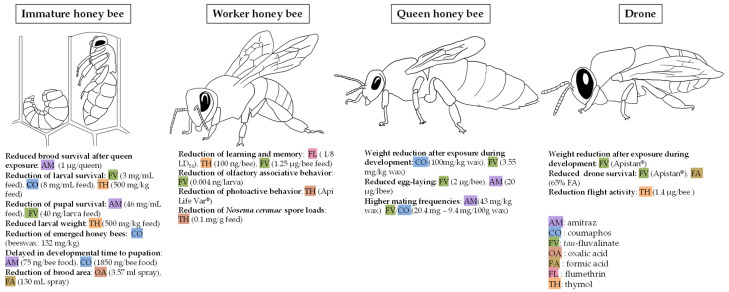
Impact of chemicals to control *V. destructor* on honey bees at the individual level. When multiple concentrations were tested in a study, only the lowest one showing a significant effect was reported. When multiple studies showed conflicting results, a significant effect was still reported if at least one study observed the significant effect.

This section presents studies investigating the impact of each compound on individual honey bees. Some endpoints have not been studied for any of the compounds. These gaps are shown in the text and in Figure 5 and are further discussed in the Conclusion.

### 7.1. Immature Honey Bees

#### 7.1.1. Brood Survival After Queen Exposure

The queen honey bee can be exposed to acaricides through nutrition or direct contact during spraying. As the queen produces female gametes and stores male gametes required for reproduction, her exposure to these chemicals may negatively affect the brood. Researchers investigated the impact of topically exposing adult queens to 1 µg of amitraz. They found that amitraz exposure significantly reduced brood survival, with significant differences observed on days 7, 9, 12, 20, and 21 (eclosion). Brood reduction ranges from 38% on day 7 to 45% on day 9 [109] (Figure 5).

#### 7.1.2. Impact on Larval Survival

Researchers have demonstrated a significant impact of acaricides used to control *V. destructor* on honey bee larval survival (Figure 5). For instance, *tau*-fluvalinate reduced the brood-capped rate by 95% when larvae were fed with 40 ng/larva [110]. Similarly, [111] reported that feeding larvae with 3 mg/mL of *tau*-fluvalinate or 8 mg/mL of coumaphos significantly decreased larval longevity. However, [112] found no significant effects on larval survival when feeding amitraz (46 mg/mL), coumaphos (25 mg/mL), or *tau*-fluvalinate (6 mg/mL). Additionally, coumaphos residues in beeswax at a concentration of 132 mg/kg significantly reduced the ratio of capped cells and emerged bees [113]. Natural chemicals, such as thymol, also impact honey bee larvae, with thymol causing a 10% increase in larval mortality when included in feed [61].

#### 7.1.3. Impact on Pupal Survival

Researchers have shown that acaricides can affect not only the longevity of honey bee larvae, but also the survival of honey bee pupae (Figure 5). When larvae were fed with 46 mg/mL of amitraz, pupal survival decreased by 40%. In contrast, no significant differences in pupal survival were observed for coumaphos (maximum concentration: 25 mg/mL) or *tau*-fluvalinate (maximum concentration: 6 mg/mL) [112]. Another group reported a significant reduction in pupal survival when larvae were fed with 40 ng of *tau*-fluvalinate per larva [110]. Additionally, another set of researchers demonstrated that the time required to reach the pupal stage was significantly delayed by exposure to amitraz (75 ng/bee) and coumaphos (1850 ng/bee), while *tau*-fluvalinate (4.59 μg/bee feed) did not cause such a delay [87].

#### 7.1.4. Impact on Immature Honey Bee External Physiology

The reduction in immature honey bee weight suggests potential alterations in nutritional behavior, indicating a negative impact of acaricides (Figure 5). A study reported a 30% decrease in larval body mass when exposed to 500 mg/kg of thymol in food, while no significant effects were observed at lower concentrations [61]. Another study also reported a significant reduction in larval size at day 3 after oral exposure to 146 ng/g of thymol. The weight of pupae and adults was not affected at this concentration, nor was the rate of development [114]. No studies, to our knowledge, have been carried out on the impact of acaricides on other external physiological aspects.

### 7.2. Adult Worker Honey Bees

#### 7.2.1. Honey Bee Learning

Odor responses play a critical role in honey bees, particularly in foraging activities. However, acaricides, due to their modes of action, can impact the nervous system of honey bees, potentially affecting learning and memory (Figure 5). Researchers have explored these effects using the proboscis extension reflex (PER) as a measure of honey bee learning. Acute exposure to 600 ppb of amitraz had no impact on honey bee learning or short-term memory as measured by PER [115]. The PER success rate of worker bees in the control group was significantly higher than that of the 1/4 LD_50_ and 1/8 LD_50_ groups after exposure to flumethrin [116]. In honey bees previously exposed to thymol (10 or 100 ng/bee), the specificity of the response to the conditioned stimulus (CS) was lost 24 h after learning [117]. However, some researchers have indicated that field-relevant exposures to acaricides may not always impair learning. For instance, [118] found no reduction in honey bee performance in a visual learning assay following *tau*-fluvalinate or thymol exposure. Some researchers found that thymol exposure did not impact odor detection at 24 h but significantly modified performance at 1 h [119]. Nevertheless, other researchers reported that high oral doses of *tau*-fluvalinate negatively affected honey bee learning, memory, sucrose responsiveness, and survival [120].

#### 7.2.2. Honey Bee Behavior

Acaricides can impact the behavior of honey bees (Figure 5). Researchers find that the olfactory associative behavior of adult bees was impaired when they were treated with sublethal doses from 0.004 to 4 ng *tau*-fluvalinate/larva in the larval stage [110]. Moreover, Api Life Var^®^ (thymol) was shown to disrupt the photoactive behavior of honey bees [121].

#### 7.2.3. Honey Bee Stressors

Honey bees can be exposed to many different stressors. Chemicals used for *V. destructor* control can have negative or positive impacts on these stressors. For instance, thymol oral supplementation has been demonstrated to reduce spore loads in adult bees infected with *Nosema ceranae* [62]. However, thymol in a 160 ppm sucrose solution did not improve the survivorship of IAPV-inoculated bees [122].

### 7.3. Queen Honey Bee

#### 7.3.1. Queen Development

Exposure to acaricides during the immature stages appears to significantly impact queen development (Figure 5). When immature queens were exposed to 100 mg/kg of coumaphos in beeswax, nearly all failed to develop, with only one queen surviving, and over 50% of cells being rejected [101]. This observation was supported by a field study, which found that queens exposed to coumaphos weighed significantly less than control queens [98]. Similar results were observed with queens exposed to high doses of *tau*-fluvalinate, which also led to reduced queen weight [98]. In honey bee queens, weight is positively correlated with spermatheca size [123]. This relationship is false under the influence of acaricides such as coumaphos and *tau-*fluvalinate, as demonstrated by [124].

#### 7.3.2. Egg Laying

Exposure appears to affect the egg-laying activity of honey bee queens (Figure 5). Colonies treated with amitraz or *tau*-fluvalinate showed a significant reduction in oviposition rates. In *tau*-fluvalinate-treated colonies, egg-laying decreased by 9.7% compared to the control group, while amitraz treatment resulted in a 7.9% reduction [125]. However, another study reported no significant difference in queen oviposition following the topical application of amitraz [109]. Additionally, formic acid, when applied in the hive at the manufacturer-recommended dose, did not significantly impact queen egg-laying activity [126].

#### 7.3.3. Mating Behavior

Acaricides have been shown to influence the mating behavior of queen honey bees (Figure 5). Queens reared in beeswax containing a combination of *tau*-fluvalinate and coumaphos, or amitraz, showed significantly higher mating frequencies [99,102].

### 7.4. Drone Honey Bee

Few studies have explored the impact of acaricides on individual drones, focusing on aspects such as longevity, external physiology, or behavior. This gap may partly be due to challenges in rearing drones under controlled experimental conditions.

#### 7.4.1. Drone Survival

Field studies indicate that Apistan^®^ (*tau*-fluvalinate) negatively affects drone survival, with 10% more drone mortality compared to the control group [104]. Similarly, exposure to formic acid has been associated with reduced drone survival rates [127] (Figure 5).

#### 7.4.2. Drone External Physiology

Apistan^®^ (*tau*-fluvalinate) also affects drone external physiology, leading to significantly lower body weights compared to the control group (Figure 5). The same study found that flight times remained unchanged [104]. Additionally, thymol has been reported to probably influence drone flight activity [44].

## 8. Impact of Acaricides at the Colony Level

**Figure 6:**
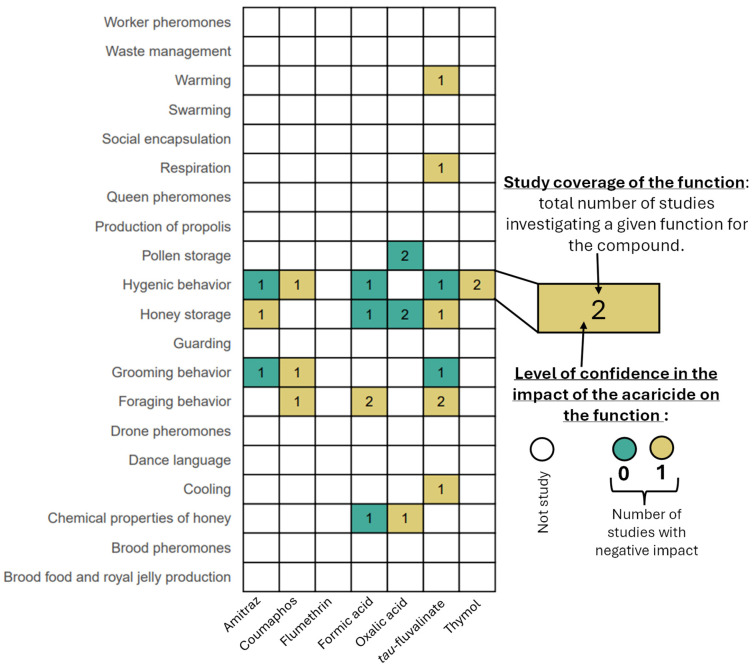
Impact of chemicals to control *V. destructor* on honey bees at the colony level. The number shown in each box corresponds to the total number of studies investigating the function in the colony for the compound. The color of the box indicates the level of confidence in the observed impact.

Honey bee colonies function as a ‘superorganism,’ with different processes similar to those of an individual organism. These include food intake, respiration, thermoregulation, immune responses, and communication. This section presents studies investigating the impact of each compound at the colony level. Some endpoints have not been studied for any of the compounds. These gaps are shown in Figure 6 and are further discussed in the Conclusion.

### 8.1. Food Collection, Processing and Consumption

Researchers have observed a reduction in honey production following treatment with some acaricides. After exposure to *tau*-fluvalinate, honey production decreased by 21.9% compared to controls, while amitraz treatment resulted in a 12.1% decrease [125]. However, no significant differences in honey production were observed after formic acid treatment [126] or after Thymovar^®^ (thymol) treatment [128]. Several recent research studies on oxalic acid have not demonstrated any impact on honey or pollen stores in the colonies [129,130].

Foraging behavior can also be impacted by acaricides. While no differences in foraging activity were observed after exposure to Apistan^®^ (*tau*-fluvalinate) or formic acid in a study [126], others reported a decrease in foraging activity following topical treatment with low doses (LD_05_) of *tau*-fluvalinate, coumaphos, and formic acid [38]. Additionally, pollen foragers made significantly fewer trips after exposure to these acaricides [38]. Acid-based acaricides may alter the properties of honey. For instance, treatment with oxalic acid significantly reduced honey pH, while formic acid had no such effect [131].

### 8.2. Colony Respiration and Thermoregulation

Acaricides can disrupt colony respiration and thermoregulation. *tau*-fluvalinate, for example, impairs wing fanning, which negatively affects temperature, humidity, and carbon dioxide levels within the hive [70].

### 8.3. Colony Immune System

The immune system of honey bee colonies partly depends on behaviors such as hygienic behaviors, grooming behavior, and social encapsulation. Coumaphos significantly impairs hygienic behavior, while *tau*-fluvalinate, amitraz, thymol, and formic acid showed no such effect [38] (Figure 6). Grooming behavior was negatively impacted by coumaphos but not by *tau*-fluvalinate or amitraz [132]. Colonies treated with Apiguard^®^ (thymol) showed an increase in the uncapping and removal of dead larvae by at least 24%, but had no effect on the removal of dead adult bees [133].

### 8.4. Worker Population

Most colony-level studies will include measurements of colony population, and acaricides certainly have demonstrated an ability to reduce worker populations under certain conditions (Figure 6). For instance, one researcher reported a one-third reduction in the adult bee population after colonies were exposed to coumaphos strips [134]. Though spraying combs with a sucrose solution containing 1% oxalic acid significantly reduced the number of adult bees in the colony [135], other researchers observed no differences in the number of adult bee frames after oxalic acid treatments using different application methods [40,129,136], unless using extremely high doses [100]. No adverse effects of Thymovar^®^ (thymol) were found on the number of adult bees in the hive up to wintering after treatment [128].

### 8.5. Brood Area

Similarly, the administration of acaricides in a colony can significantly impact the brood area following treatment (Figure 6). As most field studies include the measurement of brood area as a strength parameter, we will just highlight a few studies here. Fumigation with 12.5 mg of amitraz has been reported to reduce the brood area by 65–93% [137]. Additionally, the worker brood area decreased by 20% after two months of treatment with 130 mL of formic acid [138]. In contrast, another group found no significant effect on brood area when colonies were treated with formic acid or Apistan^®^ (*tau*-fluvalinate) [126]. However, 3.57 mL oxalic acid treatment resulted in a 22% reduction in brood area [139]. Thymovar^®^ (thymol) did not have a negative impact on the brood surface in another study [128].

### 8.6. Disease

Honey bee colonies face many stressors, and chemicals used for *V. destructor* control can have both negative and positive effects. A field study showed that feeding colonies a combination of thymol and ozone nanoparticles can be effective for treating nosemosis [140].

### 8.7. Survival of the Colony

The choice of acaricide also affects colony survival. In a comparison between oxalic acid and amitraz, oxalic acid-treated colonies showed better overwintering survival [141].

## 9. What Is the Real Sublethal Risk of Acaricides for Honey Bees?

In the hive, honey bees are exposed to various levels of chemicals to control *V. destructor* depending on the amount applied by beekeepers, the degradation rate of the chemicals, and the tasks performed by the honey bees. Researchers have previously shown that, at certain concentrations, chemicals to control *V. destructor* can have either significant or non-significant negative impacts on honey bees. The risk assessment determines whether honey bees exposed in the field encounter concentrations that have a significant impact on their behavior, reproduction, and physiology.

Comparing LOEC (Lowest Observed Effect Concentration) values with honey bee exposure can help determine whether bees are exposed to concentrations that may cause sublethal effects. All exposure values above the LOEC are defined as a risk, while all values below the LOEC are considered no risk.

**Figure 7:**
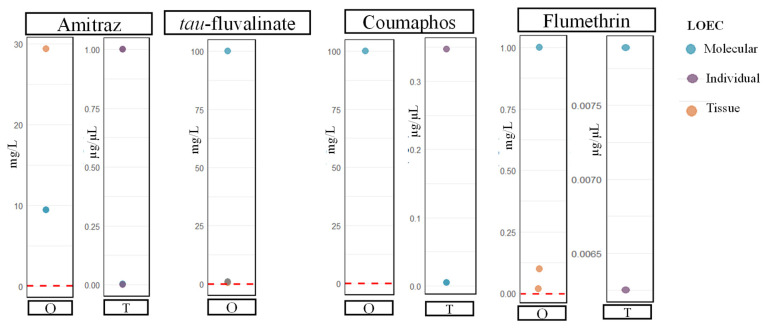
Lowest Observed Effect Concentration (LOEC) values for oral (O) or topical (T) exposure of adult honey bees reported in the studies included in this review. Points correspond to oral LOECs expressed in mg/L (O) or topical LOECs expressed in µg/µL (T). No significant differences between control and *tau*-fluvalinate-treated groups were reported in the studies reviewed, explaining why topical LOEC values for this compound are absent from the figure. The red line represents the median residue concentrations in honey [142]. To convert residue levels from µg/kg to mg/L, concentrations were multiplied by 1.42 (honey density).

By oral exposure, many studies cited in this review did not find significant sublethal impacts on honey bees at the concentrations tested. For the studies that did report significant impacts, the concentrations causing effects were mostly much higher than the median residue levels found in honey (Figure 7). Moreover, we do not have a clear understanding of how much honey bees are exposed when they contact treatment strips inside the hive. Some studies have reported residues on collected adult bees, but that is all we know at the present time. In addition, no study has analyzed residues in brood food, making it difficult to determine the risk for immature honey bees.

## 10. Summary of Impacts of Acaricides on Honey Bee Systems

### 10.1. Circulatory System

The circulatory system of the honey bee plays a role in distributing nutrients, hormones, and immune peptides throughout the organism. The composition of the hemolymph has been shown to change after exposure to both soft and synthetic acaricides, for example, in terms of the quantity of immune peptides or detoxification enzymes [45,46,47,48,49,50,51,52,53,54,55,56,57,58,59,60,61,62,63,64,65,66]. However, for these changes, it is difficult to determine their biological relevance. We do not know whether these variations truly have negative impacts on honey bees at the levels observed. At the tissue level, changes in the microbiota may also impact the composition of the hemolymph, as nutrients are transferred from the digestive system into the circulation [79,80,81,82,83]. To move within the organism, hemolymph is circulated by muscular activity, particularly the heart. However, most acaricides act on the nervous system, which is directly linked to muscle activity. Amitraz, for example, has been shown to affect heart rate and may therefore impact the circulatory system of honey bees [67,68,69]. At the individual and colony levels, there are no studies directly investigating the impact of acaricides on the circulatory system. However, some observed effects, such as reduced honey production [125], could be linked to a decrease in the overall physiological efficiency of honey bees.

### 10.2. Development System

The development system of the honey bee is important, as the impact of acaricides on development can affect colonies in the long term by producing weaker individuals. At the molecular level, tested acaricides have been shown to reduce the production of juvenile hormone [47,53], but no clear link has been established to determine whether these changes are sufficient to disrupt normal bee development. At the individual level, these effects are associated with reduced larval and pupal survival [110,111,112], as well as delays in pupation [87]. However, these survival effects are rarely linked in studies to specific tissue abnormalities or improper development, such as alterations in the nervous system of immature bees. At the colony level, these developmental impacts can result in a reduction in brood area [137,138,139].

### 10.3. Digestive System and Excretory System

The digestive system of the honey bee is one of the first tissues to come into contact with acaricides, depending on the formulation and route of exposure. For example, oxalic acid can be applied in a sucrose solution using a trickling method, where exposure occurs as bees ingest the solution and groom themselves. At the tissue level, damage can be observed in the midgut, including epithelial necrosis [74,75,76,77,78] and disruption of the gut microbiome [79,80,81,82,83]. The Malpighian tubules are also affected, showing impairment under high oxalic acid exposure [84,85]. At the individual level, weight reduction is often observed, possibly due to impaired digestive function leading to reduced nutrient uptake and assimilation [61,114].

### 10.4. Immune System

The honey bee immune system operates at social, cellular, and molecular levels and protects against a wide range of stressors. At the molecular level, immune effectors such as defensin, abaecin, hymenoptaecin, and phenoloxidase have been shown to be altered after exposure to acaricides [45,46,47,48,49,50,51,52,53,54,55,56,57,58,59,60,61,62,63,64,65,66]. However, the direction and magnitude of these responses vary across studies, and it is still unclear whether these changes are biologically significant. At the tissue level, the impact of acaricides on the fat body, at the histological level, is not well understood. In addition, the gut microbiota plays a key role in honey bee immunity and can be disrupted by exposure to acaricides [79,80,81,82,83], potentially weakening host defenses. At the individual level, the consequences of these changes for survival and resistance to other stressors remain unclear. However, some compounds appear to have beneficial effects; for example, thymol exposure has been associated with reduced *Nosema ceranae* spore loads [62]. At the colony level, immune-related behaviors can also be affected. Reductions in grooming and hygienic behavior have been reported following chemical exposure [38,132,133]. This is particularly significant because hygienic behavior is a crucial defense against *V. destructor,* one of the main reasons beekeepers use acaricide treatments. Such behavioral changes may be linked to sublethal effects on the nervous system of honey bees.

### 10.5. Muscular System

The muscular system is closely linked to the nervous system, as muscle activity is controlled by neural signals. Therefore, the effects of acaricides on the nervous system may also indirectly impact the muscular system of honey bees. At the tissue level, some acaricides have been shown to negatively affect muscle activity in bees [70,72]. Amitraz has also been shown to affect cardiac muscle function [67,68,69]. At the individual level, impairments in flight activity have been reported in drones. At the colony level, flight activity impairment changes may affect thermoregulation and respiration of the colony [70].

### 10.6. Nervous System

The nervous system is the primary target of most acaricides used to control *V. destructor* [31,32,33,34,35,36]. At the molecular level, the number of receptors, such as NMDA and GABA, can be affected by acaricide exposure. These alterations may disrupt synaptic transmission. Moreover, the targets of these treatments are present in both honey bees and *V. destructor.* However, there are differences in structure between species that may influence sensitivity to these compounds. At the individual level, a nervous system impacted by acaricides may be the cause of the impairments in learning and memory observed in some studies [115,116,117,118,119,120]. At the colony level, such effects may contribute to altered foraging activity and other behavioral changes in honey bees [38,125].

### 10.7. Reproduction System

The reproductive system of honey bees is very important for the long-term health of the colony and colony production, and problems in this system worry beekeepers greatly. At the tissue level, the reproductive organs of queens and drones are often studied. In both, some studies show a decrease in sperm count and sperm viability [98,99,100,101,102]. The weight of reproductive organs, such as ovaries and mucus glands, can also be reduced [104,105,106,107,108]. At the individual level, queens may lay fewer eggs, while drones may have lower survival and lower body weight [44,98,101,104,109,123,124,125,126,127]. At the colony level, these effects can reduce the number of workers and may threaten the survival of the colony.

### 10.8. Respiration System

The respiratory system is less frequently studied but represents a potential route of exposure for volatile acaricides such as formic acid and thymol. At the tissue level, inhibition of respiration has been reported in larvae after formic acid exposure [41]. The impact at the individual molecular level is not yet understood.

## 11. Conclusions

**Figure 8:**
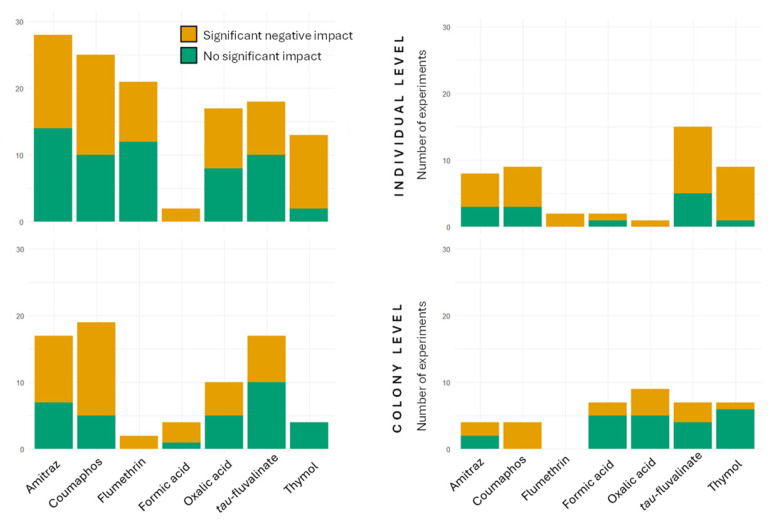
Number of experiments reporting negative and no significant effects of acaricides across biological levels. If a study includes multiple endpoints, each endpoint is considered a separate experiment. At the molecular level, each entity reported within a study is counted as one experiment. All studies included in this figure are presented in this review.

Beekeepers are strongly recommended to rotate compounds to prevent the mite from developing resistance to acaricides, but they still need to select treatments that have minimal impact on honey bee health [14]. It is commonly assumed that “soft” chemicals, including formic acid, oxalic acid, and thymol, are less harmful to honey bees. However, we did not find clear differences between synthetic and soft compounds in terms of sublethal effects on honey bees in this review (Figure 8). When comparing the number of studies reporting negative impacts with those reporting no significant effects (Figure 8), oxalic acid shows approximately equal numbers of studies in both categories across most biological levels, except at the individual level, where few studies are currently available. A similar pattern is observed for synthetic compounds such as amitraz, which shows a comparable number of studies reporting negative and no effects at the molecular and colony levels, with slightly more studies reporting negative effects at the tissue and individual levels (Figure 8).

Most studies on organic acids are conducted through colony-level exposure [60,65,83,84,107,135,139], whereas studies on synthetic compounds and thymol are more often performed under controlled laboratory conditions with defined doses [45,47,50,62,63,116,117]. Therefore, studies on organic acids may better reflect actual colony conditions, while laboratory studies on synthetic compounds and thymol may involve overexposure of bees (Figure 7). It is also important to take into account that acid treatments are hydrophilic compounds, whereas synthetic compounds and thymol are lipophilic, leading to accumulation in beeswax and potential exposure across multiple generations [17]. Interestingly, thymol shows a high proportion of studies reporting negative effects at the molecular and individual levels (Figure 8) [49,61,63,66,114,117,121,124]. In contrast, we observe a lower proportion of negative-impact studies at the tissue and colony levels [44,93,106,128,133,140]. This difference may be explained by the fact that molecular and individual studies on thymol are mostly conducted under direct laboratory exposure, whereas tissue and colony-level studies involve treatment application within colonies. Still, they highlight the importance of proper dosage and formulation, suggesting that the use of untested homemade or off-label thymol treatments may be concerning.

Among synthetic compounds, flumethrin is relatively well studied at the molecular level but less so at other biological levels, making direct comparisons more difficult (Figure 8). Among amitraz, *tau*-fluvalinate, and coumaphos, coumaphos is associated with the largest number of studies reporting negative effects, whereas *tau*-fluvalinate shows the fewest such studies across all levels (Figure 8). However, these comparisons should be interpreted with caution, as exposure doses vary between studies and may not always reflect realistic conditions (Figure 7). Perhaps most importantly, treatment efficacy must also be considered when comparing these compounds, as the benefits of effective *V. destructor* control typically outweigh potential sublethal effects. Moreover, it is important to consider all of them for rotation, as only three major modes of action are currently available.

The reproductive and developmental systems are among the most frequently studied targets of acaricides, and they are also among the most commonly reported systems with negative effects for both synthetic and soft compounds (Figure 4 and Figure 5). This is a major concern for beekeepers, as treatments may affect developing bees and reproductive individuals, weakening colonies. At the tissue level, the most studied organs include reproductive tissues such as ovaries, spermatheca, mucus glands, and seminal glands, as well as glands involved in larval nutrition, such as the hypopharyngeal glands (Figure 4). Some studies report negative effects on these tissues (Figure 4). At the individual and colony levels, numerous studies investigate impacts on brood development and queen physiology, with fewer studies focusing on drones (Figure 5). For drones, studies are mostly conducted under colony exposure [43,104,107,108,127], as they are difficult to maintain under controlled conditions. Some negative effects have been observed; however, it remains unclear whether these effects truly impact reproduction. For example, treatments such as thymol and *tau*-fluvalinate are often applied without honey supers and are mainly used in the fall, when workers naturally expel drones from the colony. As a result, affected drones may not reproduce. The level of concern regarding brood effects also depends on when treatments are applied. For instance, oxalic acid is typically used during broodless periods [14], so potential negative effects on brood observed in some studies may be less relevant under practical conditions. In contrast, synthetic treatments applied in the fall may raise greater concern if they affect brood quantity or quality, as this is the period when colonies produce winter bees that are essential for winter colony survival [143].

Many studies focus on reproduction and development in honey bees, which is understandable given their importance for beekeeping. However, it is imperative that more studies also consider the mode of action of these treatments. As most acaricides target the nervous system of mites, similar effects on honey bees may be a concern (Figure 2). Only a few studies have investigated muscle tissues in honey bees, but those that have always report negative effects of these treatments [70,72], particularly for amitraz on heart function [67,68,69]. More studies are needed to better understand these effects on colonies. In addition, the nervous system is closely linked to honey bee behavior, including foraging activity and hygienic behavior. Some studies have reported negative impacts on these behaviors [110,116,117,120,121]. Still, further research is needed, especially on behaviors that are important for colony functioning but are more difficult to study, such as dance communication and waste management.

As a precautionary approach, we will not rank treatments based on sublethal effects, as the situation is nuanced. For instance, treatments with fewer reported sublethal impacts may still cause issues for weaker colonies. Indeed, most studies are conducted on healthy colonies, and weaker colony conditions in the field may amplify observed effects. Moreover, the choice of the treatment will depend on the focus of the beekeeper. For example, queen producers may need to exercise more caution when selecting treatments that demonstrate sublethal effects on reproductive functions. Two studies on queen spermathecae have shown significant impacts following exposure to coumaphos [101,102]. In contrast, studies investigating the effects of amitraz and oxalic acid have not reported any significant impact on this tissue so far [46,99,100].

Our review highlights that dose is a key factor when considering sublethal effects of treatments on bees (Figure 5). Therefore, using registered products and strictly following label instructions (Table 1) remains the best option for beekeepers, as it ensures exposure to doses that are generally considered safe and unlikely to cause major impacts on colonies. In terms of management, colonies should only be exposed to chemical treatments when necessary to avoid unnecessary acaricide exposure. This requires monitoring mite populations and applying treatments only when infestation levels exceed thresholds [14]. Moreover, preventive management strategies should be adopted to reduce costs, labor, and potential adverse effects on colonies. In addition, treatments should be applied at the appropriate time [42], before mite populations become too high, as heavily infested colonies are often already weakened [144] and may be more sensitive to treatment adverse effects. High mite loads can also reduce treatment efficacy, potentially leading to the need for later additional treatments and, therefore, increased acaricide exposure. Another good practice for beekeepers is to rotate treatments. Rotation helps avoid the development of mite resistance, which can reduce treatment efficacy [145] and ultimately lead to increased treatment frequency.

Acaricides used for the control of *V. destructor* are incredibly beneficial for honey bee colonies and beekeepers, keeping this parasite at low levels in the hive. Acaracides are and will continue to be an essential part of responsible management regimens. However, like other mite management practices, the use of chemical compounds may be associated with potential adverse effects. It is important to understand these effects to help beekeepers recognize and manage potential adverse effects. For example, in practical terms, reproductive issues can be managed by carefully checking the queen’s laying patterns after treatment. In some cases, queens may be affected and need to be replaced. Additionally, colony recovery after treatment could potentially be supported by providing nutritional supplements.

## Data Availability

Not applicable.

## Supplementary Materials

The following supporting information can be downloaded at: https://www.mdpi.com/article/10.3390/insects17050517/s1, Table S1: Impact at the molecular level caused by major acaricides used by beekeepers for *V. destructor* control on honey bee workers; Table S2: Concentration of acaricides, time of exposure, type of application, affected area, age of bees at start of exposure, and technique used by the researchers for each reference included in Supplementary Table S1.

## Abbreviations

The following abbreviations are used in this manuscript:

ACh	Acetylcholine
AChE	Acetylcholinesterase
CS	Conditioned stimulus
CYP	Cytochrome P450
DMF	2,4-dimethylformamide
DMPF	N-(2,4-dimethylphenyl)-N′-methylformamidine
ELISA	Enzyme-linked immunosorbent assay
GABA	Gamma-aminobutyric acid
IAPV	Israeli acute paralysis virus
LC-MS	Liquid chromatography–mass spectrometry
LOEC	Lowest observed effect concentration
NMDA	N-methyl-D-aspartate
Oamb	Octopamine receptor in mushroom bodies
Oct-TyrR	Octopamine–tyramine receptor
OctαR	Octopamine alpha-adrenergic-like receptor
Octβ2R	Octopamine beta 2 receptor
PCR	Polymerase chain reaction
PER	Proboscis extension reflex
qPCR	Quantitative polymerase chain reaction
RDL	Resistant to dieldrin
TRP	Transient receptor potential
USA	United States of America

## References

[1] Aurell D., Bruckner S., Wilson M., Steinhauer N., Williams G.R. (2024). A National Survey of Managed Honey Bee Colony Losses in the USA: Results from the Bee Informed Partnership for 2020–21 and 2021–22. J. Apic. Res..

[2] Le Conte Y., Ellis M., Ritter W. (2010). *Varroa* Mites and Honey Bee Health: Can *Varroa* Explain Part of the Colony Losses?. Apidologie.

[3] Rosenkranz P., Aumeier P., Ziegelmann B. (2010). Biology and Control of *Varroa destructor*. J. Invertebr. Pathol..

[4] Vilarem C., Piou V., Vogelweith F., Vétillard A. (2021). *Varroa destructor* from the Laboratory to the Field: Control, Biocontrol and IPM Perspectives—A Review. Insects.

[5] Morfin N., Goodwin P.H., Guzman-Novoa E. (2023). *Varroa destructor* and Its Impacts on Honey Bee Biology. Front. Bee Sci..

[6] BüChler R. (1994). *Varroa* Tolerance in Honey Bees—Occurrence, Characters and Breeding. Bee World.

[7] Jack C.J., De Bem Oliveira I., Kimmel C.B., Ellis J.D. (2023). Seasonal Differences in *Varroa destructor* Population Growth in Western Honey Bee (*Apis mellifera*) Colonies. Front. Ecol. Evol..

[8] Korpela S., Aarhus A., Fries I., Hansen H. (1992). *Varroa jacobsoni* Oud. in Cold Climates: Population Growth, Winter Mortality and Influence on the Survival of Honey Bee Colonies. J. Apic. Res..

[9] Boncristiani H., Ellis J.D., Bustamante T., Graham J., Jack C., Kimmel C.B., Mortensen A., Schmehl D.R. (2021). World Honey Bee Health: The Global Distribution of Western Honey Bee (*Apis mellifera* L.) Pests and Pathogens. Bee World.

[10] Emsen B., Guzman-Novoa E., Kelly P.G. (2014). Honey Production of Honey Bee (Hymenoptera: Apidae) Colonies with High and Low *Varroa destructor* (Acari: Varroidae) Infestation Rates in Eastern Canada. Can. Entomol..

[11] Shimanuki H., Calderone N.W., Knox D.A. (1994). Parasitic Mite Syndrome: The Symptoms. Am. Bee J..

[12] Harbo J.R., Harris J.W. (2009). Responses to *Varroa* by Honey Bees with Different Levels of *Varroa* Sensitive Hygiene. J. Apic. Res..

[13] Boecking O., Genersch E. (2008). Varroosis–The Ongoing Crisis in Bee Keeping. J. Verbraucherschutz Leb..

[14] Jack C.J., Ellis J.D. (2021). Integrated Pest Management Control of *Varroa destructor* (Acari: Varroidae), the Most Damaging Pest of (*Apis mellifera* L. (Hymenoptera: Apidae)) Colonies. J. Insect Sci..

[15] Elzen P.J., Eischen F.A., Baxter J.R., Elzen G.W., Wilson W.T. (1999). Detection of Resistance in US *Varroa Jacobsoni* Oud. (Mesostigmata: Varroidae) to the Acaricide Fluvalinate. Apidologie.

[16] Spreafico M., Eördegh F.R., Bernardinelli I., Colombo M. (2001). First Detection of Strains of *Varroa destructor* Resistant to Coumaphos.Results of Laboratory Tests and Field Trials. Apidologie.

[17] Bogdanov S., Kilchenmann V., Imdorf A. (1998). Acaricide Residues in Some Bee Products. J. Apic. Res..

[18] Elzen P.J., Baxter J.R., Spivak M., Wilson W.T. (2000). Control of *Varroa jacobsoni* Oud. Resistant to Fluvalinate and Amitraz Using Coumaphos. Apidologie.

[19] Touchard A., Aili S., Fox E., Escoubas P., Orivel J., Nicholson G., Dejean A. (2016). The Biochemical Toxin Arsenal from Ant Venoms. Toxins.

[20] Keyhani J., Keyhani E. (1980). Epr Study of the Effect of Formate on Cytochrome c Oxidase. Biochem. Biophys. Res. Commun..

[21] Song C., Scharf M.E. (2008). Formic Acid: A Neurologically Active, Hydrolyzed Metabolite of Insecticidal Formate Esters. Pestic. Biochem. Physiol..

[22] Fries I., Aarhus A., Hansen H., Korpela S. (1991). Comparison of Diagnostic Methods for Detection of Low Infestation Levels of *Varroa jacobsoni* in Honey-Bee (*Apis mellifera*) Colonies. Exp. Appl. Acarol..

[23] Franceschi V.R., Nakata P.A. (2005). CALCIUM OXALATE IN PLANTS: Formation and Function. Annu. Rev. Plant Biol..

[24] Aliano N.P., Ellis M.D., Siegfried B.D. (2006). Acute Contact Toxicity of Oxalic Acid to *Varroa destructor* (Acari: Varroidae) and Their *Apis mellifera* (Hymenoptera: Apidae) Hosts in Laboratory Bioassays. J. Econ. Entomol..

[25] Gregorc A., Planinc I. (2001). Acaricidal Effect of Oxalic Acid in Honeybee (*Apis mellifera*) Colonies. Apidologie.

[26] Price K.L., Lummis S.C.R. (2022). Characterisation of Thymol Effects on RDL Receptors from the Bee Parasite *Varroa destructor*. Pestic. Biochem. Physiol..

[27] Jukic M., Politeo O., Maksimovic M., Milos M., Milos M. (2007). In Vitro Acetylcholinesterase Inhibitory Properties of Thymol, Carvacrol and Their Derivatives Thymoquinone and Thymohydroquinone. Phytother. Res..

[28] Parnas M., Peters M., Dadon D., Lev S., Vertkin I., Slutsky I., Minke B. (2009). Carvacrol Is a Novel Inhibitor of Drosophila TRPL and Mammalian TRPM7 Channels. Cell Calcium.

[29] Enan E.E. (2005). Molecular Response of *Drosophila melanogaster* Tyramine Receptor Cascade to Plant Essential Oils. Insect Biochem. Mol. Biol..

[30] Waliwitiya R., Belton P., Nicholson R.A., Lowenberger C.A. (2010). Effects of the Essential Oil Constituent Thymol and Other Neuroactive Chemicals on Flight Motor Activity and Wing Beat Frequency in the Blowfly *Phaenicia sericata*. Pest Manag. Sci..

[31] Mizunami M., Unoki S., Mori Y., Hirashima D., Hatano A., Matsumoto Y. (2009). Roles of Octopaminergic and Dopaminergic Neurons in Appetitive and Aversive Memory Recall in an Insect. BMC Biol..

[32] Farooqui T. (2012). Review of Octopamine in Insect Nervous Systems. Open Access Insect Physiol..

[33] Guo L., Fan X., Qiao X., Montell C., Huang J. (2021). An Octopamine Receptor Confers Selective Toxicity of Amitraz on Honeybees and *Varroa* Mites. eLife.

[34] Main A.R. (1979). Mode of Action of Anticholinesterases. Pharmacol. Ther..

[35] Narahashi T. (1971). Mode of Action of Pyrethroids. Bull. World Health Organ..

[36] Toshio N. (1992). Nerve Membrane Na+ Channels as Targets of Insecticides. Trends Pharmacol. Sci..

[37] Williamson S.M., Wright G.A. (2013). Exposure to Multiple Cholinergic Pesticides Impairs Olfactory Learning and Memory in Honeybees. J. Exp. Biol..

[38] Gashout H.A., Guzman-Novoa E., Goodwin P.H. (2020). Synthetic and Natural Acaricides Impair Hygienic and Foraging Behaviors of Honey Bees. Apidologie.

[39] Simon-Delso N., San Martin G., Bruneau E., Hautier L. (2018). Time-to-Death Approach to Reveal Chronic and Cumulative Toxicity of a Fungicide for Honeybees Not Revealed with the Standard Ten-Day Test. Sci. Rep..

[40] Prouty C., Abou-Shaara H.F., Stanford B., Ellis J.D., Jack C. (2023). Oxalic Acid Application Method and Treatment Intervals for Reduction of *Varroa destructor* (Mesostigmata: Varroidae) Populations in *Apis mellifera* (Hymenoptera: Apidae) Colonies. J. Insect Sci..

[41] Bolli H.K., Bogdanov S., Imdorf A., Fluri P. (1993). Zur Wirkungsweise von Ameisensäure Bei *Varroa jacobsoni* Oud Und Der Honigbiene (*Apis mellifera* L.). Apidologie.

[42] Jack C.J., Boncristiani H., Prouty C., Schmehl D.R., Ellis J.D. (2024). Evaluating the Seasonal Efficacy of Commonly Used Chemical Treatments on *Varroa destructor* (Mesostigmata: Varroidae) Population Resurgence in Honey Bee Colonies. J. Insect Sci..

[43] Fisher A., Rangel J. (2018). Exposure to Pesticides during Development Negatively Affects Honey Bee (*Apis mellifera*) Drone Sperm Viability. PLoS ONE.

[44] Johnson R.M., Dahlgren L., Siegfried B.D., Ellis M.D. (2013). Effect of In-Hive Miticides on Drone Honey Bee Survival and Sperm Viability. J. Apic. Res..

[45] Garrido P.M., Antúnez K., Martín M., Porrini M.P., Zunino P., Eguaras M.J. (2013). Immune-Related Gene Expression in Nurse Honey Bees (*Apis mellifera*) Exposed to Synthetic Acaricides. J. Insect Physiol..

[46] Chaimanee V., Pettis J.S. (2019). Gene Expression, Sperm Viability, and Queen (*Apis mellifera*) Loss Following Pesticide Exposure under Laboratory and Field Conditions. Apidologie.

[47] Ye L., Liu P., Shi T., Wang A., Zhu Y., Li L., Yu L. (2020). Transcriptomic Analysis to Elucidate the Response of Honeybees (Hymenoptera: Apidae) to Amitraz Treatment. PLoS ONE.

[48] Glavinić U., Rajković M., Ristanić M., Stevanović J., Vejnović B., Djelić N., Stanimirović Z. (2023). Genotoxic Potential of Thymol on Honey Bee DNA in the Comet Assay. Insects.

[49] Sebak S.I., Elelimy H.A.S., Seyam H., Elkenawy S.A. (2025). Effect of Thymol and Propolis Extract on Genotoxicity, Biochemical Activities and Expression Profile of Some Genes on Honey Bee, *Apis mellifera*, Infected with Vairimorpha *(Nosema*) *ceranae*. Beni Suef Univ. J. Basic Appl. Sci..

[50] Chaimanee V., Evans J.D., Chen Y., Jackson C., Pettis J.S. (2016). Sperm Viability and Gene Expression in Honey Bee Queens (*Apis mellifera*) Following Exposure to the Neonicotinoid Insecticide Imidacloprid and the Organophosphate Acaricide Coumaphos. J. Insect Physiol..

[51] Sabová L., Cingeľová Maruščáková I., Koleničová S., Mudroňová D., Holečková B., Sabo R., Sobeková A., Majchrák T., Ratvaj M. (2022). The Adverse Effects of Synthetic Acaricide *Tau*-Fluvalinate (Tech.) on Winter Adult Honey Bees. Environ. Toxicol. Pharmacol..

[52] Liu J., Shi J., Hu Y., Su Y., Zhang Y., Wu X. (2024). Flumethrin Exposure Perturbs Gut Microbiota Structure and Intestinal Metabolism in Honeybees (*Apis mellifera*). J. Hazard. Mater..

[53] Schmehl D.R., Teal P.E.A., Frazier J.L., Grozinger C.M. (2014). Genomic Analysis of the Interaction between Pesticide Exposure and Nutrition in Honey Bees (*Apis mellifera*). J. Insect Physiol..

[54] Reeves A.M., O’Neal S.T., Fell R.D., Brewster C.C., Anderson T.D. (2018). In-Hive Acaricides Alter Biochemical and Morphological Indicators of Honey Bee Nutrition, Immunity, and Development. J. Insect Sci..

[55] Cizelj I., Glavan G., Božič J., Oven I., Mrak V., Narat M. (2016). Prochloraz and Coumaphos Induce Different Gene Expression Patterns in Three Developmental Stages of the Carniolan Honey Bee (*Apis mellifera Carnica* Pollmann). Pestic. Biochem. Physiol..

[56] Zikic B., Aleksic N., Ristanic M., Glavinic U., Vejnovic B., Krnjaic I., Stanimirovic Z. (2020). Anti*-Varroa* Efficiency of Coumaphos and Its Influence on Oxidative Stress and Survival of Honey Bees. Acta Vet..

[57] Wu X., Liao C., He X., Zhang L., Yan W., Zeng Z. (2022). Sublethal Fluvalinate Negatively Affect the Development and Flight Capacity of Honeybee (*Apis mellifera* L.) Workers. Environ. Res..

[58] Liu C., Wu X., Yang H., Yu L., Zhang Y. (2022). Effects of Larval Exposure to the Insecticide Flumethrin on the Development of Honeybee (*Apis mellifera*) Workers. Front. Physiol..

[59] Qi S., Al Naggar Y., Li J., Liu Z., Xue X., Wu L., El-Seedi H.R., Wang K. (2022). Acaricide Flumethrin-Induced Sublethal Risks in Honeybees Are Associated with Gut Symbiotic Bacterium *Gilliamella apicola* through Microbe-Host Metabolic Interactions. Chemosphere.

[60] Sagona S., Tafi E., Coppola F., Nanetti A., Boni C.B., Orlando C., Palego L., Betti L., Giannaccini G., Felicioli A. (2024). Oxalic Acid Treatment: Short-Term Effects on Enzyme Activities, Vitellogenin Content, and Residual Oxalic Acid Content in House Bees, *Apis mellifera* L. Insects.

[61] Charpentier G., Vidau C., Ferdy J., Tabart J., Vetillard A. (2014). Lethal and Sub-lethal Effects of Thymol on Honeybee (*Apis mellifera*) Larvae Reared in Vitro. Pest Manag. Sci..

[62] Glavinic U., Blagojevic J., Ristanic M., Stevanovic J., Lakic N., Mirilovic M., Stanimirovic Z. (2022). Use of Thymol in *Nosema ceranae* Control and Health Improvement of Infected Honey Bees. Insects.

[63] Golob Š., Božič J., Glavan G. (2025). Potential Acaricide 2-Heptatone Induces Brain Apoptosis and Negatively Affects Survival in Honey Bees: Comparison with Thymol. Ecotoxicol. Environ. Saf..

[64] Majchrak T., Ratvaj M., Sabova L., Toporcak J., Molnar L. (2025). Toxicity of Oxalic Acid and Its Toxic Effect on Antioxidative Enzymes in Honey Bee Larvae. Veterinární Medicína.

[65] Pinďáková E., Dostálková S., Jemelková J., Fürstová J., Hurychová J., Hyršl P., Titěra D., Petřivalský M., Dobeš P., Danihlík J. (2025). Enhanced Immune Response and Antimicrobial Activity in Honey Bees (*Apis mellifera*) Following Application of Oxalic Acid-Glycerine Strips. Pestic. Biochem. Physiol..

[66] Gashout H.A., Guzman-Novoa E., Goodwin P.H., Correa-Benítez A. (2020). Impact of Sublethal Exposure to Synthetic and Natural Acaricides on Honey Bee (*Apis mellifera*) Memory and Expression of Genes Related to Memory. J. Insect Physiol..

[67] O’Neal S.T., Brewster C.C., Bloomquist J.R., Anderson T.D. (2017). Amitraz and Its Metabolite Modulate Honey Bee Cardiac Function and Tolerance to Viral Infection. J. Invertebr. Pathol..

[68] Papaefthimiou C., Papachristoforou A., Theophilidis G. (2013). Biphasic Responses of the Honeybee Heart to Nanomolar Concentrations of Amitraz. Pestic. Biochem. Physiol..

[69] Papaefthimiou C., Theophilidis G. (2011). Octopamine—A Single Modulator with Double Action on the Heart of Two Insect Species (*Apis mellifera Macedonica* and *Bactrocera oleae*): Acceleration vs. Inhibition. J. Insect Physiol..

[70] Oliver C.J., Softley S., Williamson S.M., Stevenson P.C., Wright G.A. (2015). Pyrethroids and Nectar Toxins Have Subtle Effects on the Motor Function, Grooming and Wing Fanning Behaviour of Honeybees (*Apis mellifera*). PLoS ONE.

[71] Belzunces L.P., Tchamitchian S., Brunet J.-L. (2012). Neural Effects of Insecticides in the Honey Bee. Apidologie.

[72] Arslan O.C., Erdem B., Somel M., Giray T., Kence M. (2023). Effects of Coumaphos on Locomotor Activities of Different Honeybee (*Apis mellifera* L.) Subspecies and Ecotypes. Apidologie.

[73] Yang Z., Yu Y., Zhang V., Tian Y., Qi W., Wang L. (2015). Octopamine Mediates Starvation-Induced Hyperactivity in Adult *Drosophila*. Proc. Natl. Acad. Sci. USA.

[74] Gregorc A., Alburaki M., Rinderer N., Sampson B., Knight P.R., Karim S., Adamczyk J. (2018). Effects of Coumaphos and Imidacloprid on Honey Bee (Hymenoptera: Apidae) Lifespan and Antioxidant Gene Regulations in Laboratory Experiments. Sci. Rep..

[75] Qi S., Niu X., Wang D.H., Wang C., Zhu L., Xue X., Zhang Z., Wu L. (2020). Flumethrin at Sublethal Concentrations Induces Stresses in Adult Honey Bees (*Apis mellifera* L.). Sci. Total Environ..

[76] Gregorc A., Bowen I.D. (2000). Histochemical characterization of cell death in honeybee larvae midgut after treatment with *Paenibacillus larvae*, amitraz and oxytetracycline. Cell Biol. Int..

[77] Gregorc A., Pogacnik A., Bowen I.D. (2004). Cell Death in Honeybee (*Apis mellifera*) Larvae Treated with Oxalic or Formic Acid. Apidologie.

[78] Gregorc A., Smodiš Škerl M.I. (2007). Toxicological and Immunohistochemical Testing of Honeybees after Oxalic Acid and Rotenone Treatments. Apidologie.

[79] Yang Y., Ma S., Yan Z., Liu F., Diao Q., Dai P. (2019). Effects of Three Common Pesticides on Survival, Food Consumption and Midgut Bacterial Communities of Adult Workers *Apis cerana* and *Apis mellifera*. Environ. Pollut..

[80] Żebracka A., Chmielowiec-Korzeniowska A., Nowakowicz-Dębek B., Wlazło Ł., Dziechciarz P., Borsuk G. (2022). Intestinal Microbiota of Honey Bees (*Apis mellifera*) Treated with Amitraz. J. Apic. Sci..

[81] Liu J., Liao C., Li Z., Shi X., Wu X. (2024). Synergistic Resistance of Honeybee (*Apis mellifera*) and Their Gut Microorganisms to Fluvalinate Stress. Pestic. Biochem. Physiol..

[82] Kakumanu M.L., Reeves A.M., Anderson T.D., Rodrigues R.R., Williams M.A. (2016). Honey Bee Gut Microbiome Is Altered by In-Hive Pesticide Exposures. Front. Microbiol..

[83] Gorrochategui-Ortega J., Muñoz-Colmenero M., Galartza E., Estonba A., Zarraonaindia I. (2025). Colonies under Dysbiosis Benefit from Oxalic Acid Application: The Role of Landscape and Beekeeping Practices in Microbiota Response to Treatment. J. Pest Sci..

[84] Martín-Hernández R., Higes M., Pérez J.L., Nozal M.J., Gómez L., Meana A. (2007). Short Term Negative Effect of Oxalic Acid in *Apis mellifera Iberiensis*. Span. J. Agric. Res..

[85] Papežíková I., Palíková M., Kremserová S., Zachová A., Peterová H., Babák V., Navrátil S. (2017). Effect of Oxalic Acid on the Mite *Varroa Destructor* and Its Host the Honey Bee *Apis mellifera*. J. Apic. Res..

[86] Moon S.E. (2020). The Effects of DMPF on Honey Bee Pathophysiology.

[87] Tomé H.V.V., Schmehl D.R., Wedde A.E., Godoy R.S.M., Ravaiano S.V., Guedes R.N.C., Martins G.F., Ellis J.D. (2020). Frequently Encountered Pesticides Can Cause Multiple Disorders in Developing Worker Honey Bees. Environ. Pollut..

[88] Risse M., Dainat B., Jeker L. (2018). Sub-Lethal Effects at Stake: Does the Acaricide Coumaphos and Fungicide Folpet Affect the Hypopharyngeal Glands Size?. Julius Kühn Archiv.

[89] Smodiš Škerl M.I., Kmecl V., Gregorc A. (2010). Exposure to Pesticides at Sublethal Level and Their Distribution Within a Honey Bee (*Apis mellifera*) Colony. Bull. Environ. Contam. Toxicol..

[90] Smodiš Škerl M.I., Gregorc A. (2010). Heat Shock Proteins and Cell Death in Situ Localisation in Hypopharyngeal Glands of Honeybee ( *Apis mellifera Carnica* ) Workers after Imidacloprid or Coumaphos Treatment. Apidologie.

[91] Weisbrod J.M. (2020). Effects of Pesticide Residue Accumulation on Honey Bee (*Apis mellifera* L.) Development and Implications for Hive Management. Master’s Thesis.

[92] Salem R.A., El-sayıed H.M., Amro A., Abd Alla A. (2024). Impact of acaricides on *Varroa destructor* infestation in honey bee colonies (*Apis mellifera* L.) and their histological effects on hypopharyngeal glands. Uludağ Arıcılık Derg..

[93] Aboushaara H., Staron M., Čermáková T. (2017). Impacts of Oxalic Acid, Thymol, and Potassium Citrate as *Varroa* Control Materials on Some Parameters of Honey Bees. Turk. J. Vet. Anim. Sci..

[94] Slessor K.N., Kaminski L.-A., King G.G.S., Borden J.H., Winston M.L. (1988). Semiochemical Basis of the Retinue Response to Queen Honey Bees. Nature.

[95] Walsh E.M., Sweet S., Knap A., Ing N., Rangel J. (2020). Queen Honey Bee (*Apis mellifera*) Pheromone and Reproductive Behavior Are Affected by Pesticide Exposure during Development. Behav. Ecol. Sociobiol..

[96] Nolan W.J. (1925). The Brood-Rearing Cycle of the Honeybee.

[97] Walsh E.M., Khan O., Grunseich J., Helms A.M., Ing N.H., Rangel J. (2021). Pesticide Exposure During Development Does Not Affect the Larval Pheromones, Feeding Rates, or Morphology of Adult Honey Bee (*Apis mellifera*) Queens. Front. Ecol. Evol..

[98] Haarmann T., Spivak M., Weaver D., Weaver B., Glenn T. (2002). Effects of Fluvalinate and Coumaphos on Queen Honey Bees (Hymenoptera: Apidae) in Two Commercial Queen Rearing Operations. J. Econ. Entomol..

[99] Walsh E.M., Janowiecki M.A., Zhu K., Ing N.H., Vargo E.L., Rangel J. (2021). Elevated Mating Frequency in Honey Bee (Hymenoptera: Apidae) Queens Exposed to the Miticide Amitraz During Development. Ann. Entomol. Soc. Am..

[100] Tellarini Prieto E.E., Pietropaoli M., Camus Y., Polizel Camilli M., Raza M.F., Jose M.S., Obshta O., Bezerra Da Silva M.C., Kozii I., Moshynskyy I. (2024). Safety Assessment of High Doses of Vaporized Oxalic Acid on Honey Bee Worker Health and Queen Quality. Front. Bee Sci..

[101] Collins A.M., Pettis J.S., Wilbanks R., Feldlaufer M.F. (2004). Performance of Honey Bee (*Apis mellifera*) Queens Reared in Beeswax Cells Impregnated with Coumaphos. J. Apic. Res..

[102] Rangel J., Tarpy D.R. (2015). The Combined Effects of Miticides on the Mating Health of Honey Bee (*Apis mellifera* L.) Queens. J. Apic. Res..

[103] Hayashi S., Satoh T. (2019). Sperm Maturation Process Occurs in the Seminal Vesicle Following Sperm Transition from Testis in Honey Bee Males. Apidologie.

[104] Rinderer T., De Guzman L., Lancaster V., Stelzer A. (1998). *Varroa* in the Mating Yard: I. The Effects of *Varroa jacobsoni* and Apistan on Drone Honey Bee. Am. Bee J..

[105] Delaney D.A. (2003). Consequences of Coumaphos and *Varroa destructor* on Drone Honey Bee Sperm Quantity. Master’s Thesis.

[106] Shoukry R.S., Khattaby A.M., El-Sheakh A.A., Abo-Ghalia A.H., Elbanna S.M. (2013). Effect of some materials for controlling *varroa* mite on the honeybee drones (*Apis mellifera*) L. *Egypt*. J. Agric. Res..

[107] Ben Abdelkader F., Çakmak İ., Çakmak S.S., Nur Z., İncebıyık E., Aktar A., Erdost H. (2021). Toxicity Assessment of Chronic Exposure to Common Insecticides and Bee Medications on Colony Development and Drones Sperm Parameters. Ecotoxicology.

[108] Burley L.M., Fell R.D., Saacke R.G. (2008). Survival of Honey Bee (Hymenoptera: Apidae) Spermatozoa Incubated at Room Temperature from Drones Exposed to Miticides. J. Econ. Entomol..

[109] Kayode A., Lizette D., Johnson R., Siegfried B.D., Ellis M. (2014). Effect of Amitraz on Queen Honey Bee Egg and Brood Development.

[110] Ko C.-Y., Nai Y.-S., Lo W., Chen C.-T., Chen Y.-W. (2022). Low-Level Fluvalinate Treatment in the Larval Stage Induces Impaired Olfactory Associative Behavior of Honey Bee Workers in the Field. Insects.

[111] Zhu W., Schmehl D.R., Mullin C.A., Frazier J.L. (2014). Four Common Pesticides, Their Mixtures and a Formulation Solvent in the Hive Environment Have High Oral Toxicity to Honey Bee Larvae. PLoS ONE.

[112] Dai P., Jack C.J., Mortensen A.N., Bustamante T.A., Ellis J.D. (2018). Chronic Toxicity of Amitraz, Coumaphos and Fluvalinate to *Apis mellifera* L. Larvae Reared in Vitro. Sci. Rep..

[113] Kast C., Droz B., Kilchenmann V. (2023). Toxicity of Coumaphos Residues in Beeswax Foundation to the Honey Bee Brood. Environ. Toxicol. Chem..

[114] Yordanova M., Zhang X., Torres C.B., Evison S.E.F., Gill R.J., Graystock P. (2025). Friend or Foe? Concentration of a Commensal Microbe Induces Distinct Responses in Developing Honey Bees Exposed to Field-Realistic Pesticide Concentrations. FEMS Microbiol. Ecol..

[115] Rix R.R., Christopher Cutler G. (2016). Acute Exposure to Worst-Case Concentrations of Amitraz Does Not Affect Honey Bee Learning, Short-Term Memory, or Hemolymph Octopamine Levels. J. Econ. Entomol..

[116] Wu X., Li Z., Yang H., He X., Yan W., Zeng Z. (2023). The Adverse Impact on Lifespan, Immunity, and Forage Behavior of Worker Bees (*Apis mellifera* Linnaeus 1758) after Exposure to Flumethrin. Sci. Total Environ..

[117] Bonnafé E., Drouard F., Hotier L., Carayon J.-L., Marty P., Treilhou M., Armengaud C. (2015). Effect of a Thymol Application on Olfactory Memory and Gene Expression Levels in the Brain of the Honeybee *Apis mellifera*. Environ. Sci. Pollut. Res..

[118] Colin T., Plath J.A., Klein S., Vine P., Devaud J.-M., Lihoreau M., Meikle W.G., Barron A.B. (2020). The Miticide Thymol in Combination with Trace Levels of the Neonicotinoid Imidacloprid Reduces Visual Learning Performance in Honey Bees (*Apis mellifera*). Apidologie.

[119] Chapuy C., Ribbens L., Renou M., Dacher M., Armengaud C. (2019). Thymol Affects Congruency Between Olfactory and Gustatory Stimuli in Bees. Sci. Rep..

[120] Frost E.H., Shutler D., Hillier N.K. (2013). Effects of Fluvalinate on Honey Bee Learning, Memory, Responsiveness to Sucrose, and Survival. J. Exp. Biol..

[121] Carayon J.-L., Téné N., Bonnafé E., Alayrangues J., Hotier L., Armengaud C., Treilhou M. (2014). Thymol as an Alternative to Pesticides: Persistence and Effects of Apilife Var on the Phototactic Behavior of the Honeybee *Apis mellifera*. Environ. Sci. Pollut. Res..

[122] Hsieh E.M., Berenbaum M.R., Dolezal A.G. (2020). Ameliorative Effects of Phytochemical Ingestion on Viral Infection in Honey Bees. Insects.

[123] Akyol E., Yeninar H., Kaftanoglu O. (2008). Live Weight of Queen Honey Bees (*Apis mellifera* L.) Predicts Reproductive Characteristics. J. Kans. Entomol. Soc..

[124] Collins A.M., Pettis J.S. (2013). Correlation of Queen Size and Spermathecal Contents and Effects of Miticide Exposure during Development. Apidologie.

[125] Ilyasov R., Lim S., Lee M.L., Kwon H.W., Nikolenko A. (2021). Effect of miticides amitraz and fluvalinate on reproduction and productivity of honey bee *Apis mellifera*. Uludağ Arıcılık Derg..

[126] Westcott L.C., Winston M.L. (1999). Chemical acaricides in *Apis mellifera* (hymenoptera: Apidae) colonies; do they cause nonlethal effects?. Can. Entomol..

[127] De Guzman L., Rinderer T., Lancaster V., Delatte G., Stelzer A. (1999). *Varroa* in the Mating Yard: III. The Effects of Formic Acid Gel Formulation On drone production. Am. Bee J..

[128] Manzano Sánchez L., Gómez Ramos M.J., Gómez-Ramos M.D.M., Parrilla Vazquez P., Flores J.M., Fernández-Alba A.R. (2021). Presence, Persistence and Distribution of Thymol in Honeybees and Beehive Compartments by High Resolution Mass Spectrometry. Environ. Adv..

[129] Jack C.J., Van Santen E., Ellis J.D. (2021). Determining the Dose of Oxalic Acid Applied via Vaporization Needed for the Control of the Honey Bee (*Apis mellifera*) Pest *Varroa destructor*. J. Apic. Res..

[130] Berry J.A., Bartlett L.J., Bruckner S., Baker C., Braman S.K., Delaplane K.S., Williams G.R. (2022). Assessing Repeated Oxalic Acid Vaporization in Honey Bee (Hymenoptera: Apidae) Colonies for Control of the Ectoparasitic Mite *Varroa destructor*. J. Insect Sci..

[131] Borsuk G., Olszewski K., Paleolog J., Strachecka A., Gryzińska M. (2012). The Effect of Different Varroacides on the Acidity of Winter Stores and Honey Stores. Ann. UMCS Zootech..

[132] De Mattos I.M., Soares A.E.E., Tarpy D.R. (2017). Effects of Synthetic Acaricides on Honey Bee Grooming Behavior against the Parasitic *Varroa destructor* Mite. Apidologie.

[133] Colin T., Lim M.Y., Quarrell S.R., Allen G.R., Barron A.B. (2019). Effects of Thymol on European Honey Bee Hygienic Behaviour. Apidologie.

[134] Gregorc A. (2012). A Clinical Case of Honey Bee Intoxication after Using Coumaphos Strips against *Varroa destructor*. J. Apic. Res..

[135] Toomemaa K., Martin A.-J., Williams I.H. (2010). The Effect of Different Concentrations of Oxalic Acid in Aqueous and Sucrose Solution on *Varroa* Mites and Honey Bees. Apidologie.

[136] Kulhanek K., Hopkins B.K., Sheppard W.S. (2023). Comparison of Oxalic Acid Drip and HopGuard for Pre-Winter *Varroa destructor* Control in Honey Bee (*Apis mellifera*) Colonies. J. Apic. Res..

[137] Pohorecka K., Skubida P., Semkiw P. (2018). Varroacidal Efficiency of Treatment with Amitraz in Honey Bee Colonies with Brood. J. Apic. Sci..

[138] Ostermann D.J., Currie R.W. (2004). Effect of Formic Acid Formulations on Honey Bee (Hymenoptera: Apidae) Colonies and Influence of Colony and Ambient Conditions on Formic Acid Concentration in the Hive. J. Econ. Entomol..

[139] Hatjina F., Haristos L. (2005). Indirect Effects of Oxalic Acid Administered by Trickling Method on Honey Bee Brood. J. Apic. Res..

[140] Özüiçli M., Aydin L., Girişgin A.O., Selova S., Sabanci A.Ü. (2024). Determination of the Efficacy of Thymol, *Artemisia absinthium* Oil and Nanoparticle Ozone in the Treatment of *Nosema ceranae* in Adult Honey Bees. J. Apic. Res..

[141] Papežíková I., Palíková M., Navrátil S., Heumannová R., Fronc M. (2016). The Effect of Oxalic Acid Applied by Sublimation on Honey Bee Colony Fitness: A Comparison with Amitraz. Acta Vet. Brno.

[142] El-Nahhal Y. (2020). Pesticide Residues in Honey and Their Potential Reproductive Toxicity. Sci. Total Environ..

[143] Mattila H.R., Harris J.L., Otis G.W. (2001). Timing of Production of Winter Bees in Honey Bee (*Apis mellifera*) Colonies. Insectes Sociaux.

[144] Barroso-Arévalo S., Fernández-Carrión E., Goyache J., Molero F., Puerta F., Sánchez-Vizcaíno J.M. (2019). High Load of Deformed Wing Virus and *Varroa destructor* Infestation Are Related to Weakness of Honey Bee Colonies in Southern Spain. Front. Microbiol..

[145] Rinkevich F.D. (2020). Detection of Amitraz Resistance and Reduced Treatment Efficacy in the *Varroa* Mite, *Varroa destructor*, within Commercial Beekeeping Operations. PLoS ONE.

