# Supplementary material for: Sublethal Effects and Associated Risks of Acaricides Used Against Varroa destructor in Honey Bee (Apis mellifera) Colonies
Source: Insects. 2026 May 19;17(5):517. doi: 10.3390/insects17050517 (PMC13206983; doi:10.3390/insects17050517)
Supplement: Supplementary file 1 [file insects-17-00517-s001.zip › insects-4273507-supplementary.pdf]

Table S1. Impact at the molecular level caused by major acaricides used by beekeepers for *V. destructor* control on honey bee workers (↑ : significant increase compared to the control (no treatment); ↓: significant decrease compared to the control (no treatment); →: no significant increase or decrease compared to the control (no treatment)).

Biological process	Name	Amitraz	Coumaphos	*tau*-fluvalinate	Flumethrin	Oxalic acid	Formic acid	Thymol
**Immune sytem/behavior**	Vitellogenin	→[a] ↑[c] ↓[i]	→[a]	→[a]	→[a] ↑[u]	↑[o] ↑[u]		↑[p]
**Immune system**	Lysozyme	→[a]	→[a] ↑[h]	→[a]	→[a]	→[t]		
**Immune system**	Glucose dehydrogenase	→[a] ↑[c]	→[a]	→[a]	→[a]			
**Immune system**	Phenoloxidase	→[a]	→[a] →[g]	→[a] →[g]	→[a]	→[o]		
**Immune system**	Defensin	→[a] →[i] ↓[v]	→[a] ↓[h] ↓[v]	→[a] ↓[d] ↓[v]	→[a] ↑[m]	↑[t] ↑[u]	↓[v]	↑[s] ↓[v]
**Immune system**	Abaecin	→[a] →[i]	→[a] ↓[h]	→[a]	→[a] →[m]	↑[u]		↑[s]
**Immune system**	Hymenoptaecin	→[a] →[i]	→[a]	→[a] ↓[d]	↑[a]	→[t] ↑[u]		↑[s]
**Immune system**	Apidaecin	↑[c] →[i]	→[b]		→[m]	↑[u]		
**Detoxification**	CYP306A1	→[i]	↑[b]					
**Detoxification**	CYP4G11	→[i]	↓[b]					
**Stress**	Superoxide dismutase	↓[i]	↑[b] ↓[j]	↑[k]	↓[e]	→[t]		↓[s]
**Stress**	Thioredoxin peroxidase	↓[i]	↑[b]					
**Storage protein**	Hexamerin 70b	↓[i]	↑[b]					
**Detoxification**	Glutathione S-transferase	→[i]	↑[j]	→[k]	→[d] ↓[e] ↑[l]	→[o] ↑[t]		→[r] ↓[s]
**Nervous system**	Glutamate receptors	↑[c]			↓[d]			
**Behavior**	Tyramine				→[d]			
**Nervous system**	NMDA receptors				→[d]			
**Detoxification**	CYP450	↑[c]		↑[k]	→[d]			
**Stress**	Catalase	↓[e] ↓[i]	↓[e] ↑[j]			→[o] →[t]		↓[s]
**Development**	Juveline hormone and percursors*	↓[c]	↓[f]	↓[f]				
**Antibacterial activity**	Glucose oxidase		↑[g]	↑[g]		→[o]		
**Detoxification**	Carboxylic acid esterase			→[k]	↑[l]			
**Olfactory system**	Odorant binding protein				↓[l]			
**Stress**	Malondialdehyde				→[u]	↓[u]		↑[s]
**Development**	Major Royal Jelly Proteins	↓[v]	→[v]	↓[v]			↓[v]	↑[s] →[s] ↓[v]

Table S2. Concentration of acaricides, time of exposure, type of application, affected area, age of bees at start of exposure, and technique used by the researchers for each reference included in Supplementary_Table_1.

Study	Concentration of acaricides	Time of exposure	Type of application	Affected area	Age of bees at start exposure	Technique used
**a [45]**	Flumethrin (7.89 ppm); *tau*-fluvalinate (61–43 ppm); coumaphos (751.07 ppm); amitraz (282.91 ppm)	24 h	Topical	Entire honey bee	6-day-old	Real-time PCR
**b [50]**	Coumaphos (5–100 ppm)	7 days	Topical	Entire honey bee	2-day-old	Real-time PCR
**c [47]**	Amitraz (9.4 mg/L)	10 days	Oral	Midgut	3-day-old	Transcriptomic analysis
**d [51]**	*Tau*-fluvalinate (750 µg/kg)	10 days	Oral	Gut	3-day-old	Real-time PCR
**e [52]**	Flumethrin (0.01–1 mg/L)	14 days	Oral	Intestine	1-day-old	ELISA
**f [53]**	Coumaphos (100 ppm); *tau*-fluvalinate (100 ppm)	7 days	Oral	Hemolymph	2-day-old	Enzymatic assays
**g [54]**	Coumaphos (CheckMite+®); *tau*-fluvalinate (Apistan®)	6 weeks	In hive	Entire honey bee	Nurse–forager bees	Enzymatic assays
**h [55]**	Coumaphos (Perizin®)	10 days	In hive	Entire honey bee	Prepupa–adult	Real-time PCR
**i [46]**	Amitraz (2.5 ppm)	24 h	Topical	Abdomen	Queen bee	Real-time PCR
**j [56]**	Coumaphos (CheckMite+®)	42 days	In hive	Entire honey bee	Not specified (X)	Enzymatic assays
**k [57]**	*Tau*-fluvalinate (0.5–50 mg/kg)	4 days	Oral	Entire larvae	6-day-old	Enzymatic assays
**l [58]**	Flumethrin (0.01–1 mg/L)	5 days	Oral	Entire larvae	7-day-old	Enzymatic assays
**m [59]**	Flumethrin (10 µg/L)	14 days	Oral	Midgut	2-day-old	Real-time PCR
**o [60]**	Oxalic acid (Api-Bioxal®)	48 h	In hive	Head	Not specified (X)	Enzymatic assays
**p [61]**	Thymol (1–3000 mg/kg food)	6 days	Oral	Entire larvae	2-day-old	Real-time PCR

**q [62]**	Thymol (0.1 g/kg)	9 days	Oral	Entire honey bee	2-day-old	Enzymatic assays
**r [63]**	Thymol (12.5–50 ppm)	5 days	Oral	Head and thorax	2-day-old	Enzymatic assays
**s [49]**	Thymol (0.1 g/L in colonies)	Not specified (X)	Oral	Entire honey bee	Not specified (X)	qPCR and enzymatic assays
**t [64]**	Oxalic acid (0–7%)	4 days	Oral	Entire larva	Not specified (X)	qPCR
**u [65]**	Oxalic acid (glycerine strips/trickling); flumethrin (Gabon)	Not specified (X)	Not specified (X)	Not specified (X)	Not specified (X)	LC-MS; enzymatic assays
**v [66]**	*Tau*-fluvalinate; amitraz; coumaphos; thymol; formic acid (LD05 or LD50)	24 h	Topical	Entire honey bee	19-day-old	RT-PCR
